# Extracellular vesicles from bodily fluids for the accurate diagnosis of Parkinson's disease and related disorders: A systematic review and diagnostic meta‐analysis

**DOI:** 10.1002/jex2.121

**Published:** 2023-11-13

**Authors:** Hash Brown Taha, Aleksander Bogoniewski

**Affiliations:** ^1^ Department of Integrative Biology & Physiology University of California Los Angeles Los Angeles California USA; ^2^ Department of Molecular and Medical Pharmacology, David Geffen School of Medicine University of California Los Angeles Los Angeles California USA

**Keywords:** alpha‐synuclein, biomarker, exosome, L1CAM, movement disorders, parkinsonism

## Abstract

Parkinsonian disorders, including Parkinson's disease (PD), multiple system atrophy (MSA), dementia with Lewy body (DLB), corticobasal syndrome (CBS) and progressive supranuclear palsy (PSP) are often misdiagnosed due to overlapping symptoms and the absence of precise biomarkers. Furthermore, there are no current methods to ascertain the progression and conversion of prodromal conditions such as REM behaviour disorder (RBD). Extracellular vesicles (EVs), containing a mixture of biomolecules, have emerged as potential sources for parkinsonian diagnostics. However, inconsistencies in previous studies have left their diagnostic potential unclear. We conducted a meta‐analysis, following PRISMA guidelines, to assess the diagnostic accuracy of general EVs isolated from various bodily fluids, including cerebrospinal fluid (CSF), plasma, serum, urine or saliva, in differentiating patients with parkinsonian disorders from healthy controls (HCs). The meta‐analysis included 21 studies encompassing 1285 patients with PD, 24 with MSA, 105 with DLB, 99 with PSP, 101 with RBD and 783 HCs. Further analyses were conducted only for patients with PD versus HCs, given the limited number for other comparisons. Using bivariate and hierarchal receiver operating characteristics (HSROC) models, the meta‐analysis revealed moderate diagnostic accuracy in distinguishing patients with PD from HCs, with substantial heterogeneity and publication bias. The trim‐and‐fill method revealed at least two missing studies with null or low diagnostic accuracy. CSF‐EVs showed better overall diagnostic accuracy, while plasma‐EVs had the lowest performance. General EVs demonstrated higher diagnostic accuracy compared to CNS‐originating EVs, which are more time‐consuming, labour‐ and cost‐intensive to isolate. In conclusion, while holding promise, utilizing biomarkers in general EVs for PD diagnosis remains unfeasible due to existing challenges. The focus should shift toward harmonizing the field through standardization, collaboration, and rigorous validation. Current efforts by the International Society For Extracellular Vesicles (ISEV) aim to enhance the accuracy and reproducibility of EV‐related research through rigor and standardization, aiming to bridge the gap between theory and practical clinical application.

## 1 INTRODUCTION

Motor symptoms such as slowness of movement (bradykinesia), stiffness (rigidity), and shaking (tremor) are hallmarks of a collection of neurodegenerative disorders known as parkinsonian disorders. Among these, Parkinson's disease (PD) is the most prevalent (Poewe et al., 2017). Other notable but rarer conditions in this group are multiple system atrophy (MSA), dementia with Lewy bodies (DLB), progressive supranuclear palsy (PSP), and corticobasal syndrome (CBS) (Armstrong & Okun, 2020). Although these diseases are characterized by distinct pathophysiologies with differences in the proteins involved, affected cells, and brain regions, they are often misdiagnosed by neurologists and movement disorder specialists due to symptom overlap and lack of precise biomarkers (Surguchov, 2022), especially in the early stages (Baumann, 2012; Rizzo et al., 2016; Schrag et al., 2002). Moreover, we currently cannot predict the onset of the prodromal conditions known as rapid eye movement (REM) behaviour disorder (RBD) and/or pure autonomic failure (PAF), nor their progression and conversion into synucleinopathies such as PD, MSA and/or DLB (Dauvilliers et al., 2018). Unfortunately, a definitive diagnosis can only be obtained through a postmortem neuropathological examination after the patients have passed away.

Such incorrect diagnoses can carry profound consequences for patients, resulting not only in improper treatment and a deterioration of overall health but also hindering the identification of disease‐modifying therapies (Surguchov, 2022). This confusion can escalate patients' emotional distress, exacerbating feelings of uncertainty and anxiety regarding their medical situation.

Extracellular vesicles (EVs) are small, lipid‐bilayer‐bound entities secreted by cells, instrumental in mediating intercellular communication and orchestrating various physiological functions (Dixson et al., 2023; Surguchev et al., 2019).EVs encompass a heterogeneous mixture of biomolecules, including proteins, lipids, and nucleic acids, reflective of the state of the parent cell (Dixson et al., 2023). As such they have been widely used as a rich source for biomarker discovery (Simeone et al., 2020).

Numerous research groups have analysed biomarkers in general bodily fluid‐isolated EVs (Upadhya & Shetty, 2021) or central nervous system (CNS)‐originating EVs (Dutta et al., 2023) to differentiate parkinsonian disorders among each other or from healthy controls (HCs). However, consistent failure has been observed in independent validations and replications, leading to varying outcomes even when identical methodologies are applied.

A number of meta‐analyses have investigated the utilization of biomarkers in general EVs for differentiating patients with PD from HCs (Nila et al., 2022) or CNS‐originating EVs for distinguishing various parkinsonian disorders from one another or from HCs (Taha & Ati, 2023). These studies suggested the potential for elevated concentrations of EVs‐associated α‐synuclein in patients with PD versus HCs (Nila et al., 2022; Taha & Ati, 2023).

Additionally, a recent meta‐analysis assessed the diagnostic accuracy of biomarkers in CNS‐originating EVs for parkinsonian disorders and identified significant heterogeneity, variance, inconsistencies, and evidence of substantial publication bias (Taha & Bogoniewski, 2023). However, to date, no research has explored the diagnostic accuracy of biomarkers in general EVs for parkinsonian disorders.

Hence, we conducted a comprehensive systematic review and diagnostic meta‐analysis, incorporating all studies aimed at distinguishing parkinsonian disorders among each other or from HCs by utilizing biomarkers in general EVs isolated from bodily fluids. The analysis involves a comparison of results based on the fluid from which the EVs were isolated such as cerebrospinal fluid (CSF), plasma, serum, urine, and saliva. Lastly, we compared the diagnostic accuracy obtained from general EVs with the accuracy reported earlier for CNS‐originating EVs isolated from plasma or serum (Taha & Bogoniewski, 2023).

## 2 METHODOLOGY

We conducted a systematic review and meta‐analysis in accordance with the guidelines stipulated by the Preferred Reporting Items for Systematic Reviews and Meta‐Analyses Protocols (PRISMA). We restricted our research to the use of anonymized data, without collecting any personal information or involving human subjects, thereby negating the necessity for ethical approval. The protocol for this study was not registered.

### 2.1 Data sources and search strategy

We carried out an exhaustive search of pertinent articles using targeted search terms connected to PD and other parkinsonian disorders. This search was performed within two databases, PUBMED and EMBASE, and included articles published from the beginning of these databases up to 5 August 2023. Our search terms comprised combinations such as “Parkinson's disease OR multiple system atrophy OR Lewy body dementia OR corticobasal syndrome OR progressive supranuclear palsy” AND “Extracellular Vesicle OR exosome” AND “Diagnosis”. To identify appropriate studies for inclusion, we scrutinized the reference lists of qualifying studies and performed in‐depth literature reviews. Any disagreements regarding the selection of articles were settled through dialogue. The detailed search strategy is presented in Table S1.

### 2.2 Eligibility criteria

The studies we included in our analysis were specifically centered on evaluating biomarker levels in general EVs isolated from CSF, plasma, serum, urine, or saliva in patients with PD and at least one of the following conditions: MSA, DLB, PSP, CBS, RBD or HCs. These studies had to have performed receiver operating characteristic (ROC) analysis and provided details on sensitivity, specificity, area under curve (AUC), and sample size. We excluded any research involving animals or cell lines, those that did not focus on the specified diseases, and those that failed to report the sample size. If crucial details such as sensitivity, specificity, or sample size were missing from the study, we reached out to the authors to acquire the necessary information. In cases where studies contained longitudinal measurements or treatment interventions, we only considered the initial baseline evaluations. In cases where studies contained discovery and validation cohorts, we only considered the validation cohort.

### 2.3 Risk of bias assessment

All the qualifying studies were examined for quality and potential bias according to the Quality Assessment for Diagnostic Accuracy Studies (QUADAS‐2) standards (Whiting et al., 2011). This evaluation was conducted by independent researchers (H.B.T. and A.B.), and in cases of disagreement, discussions were held until an agreement was reached. Further specifics about the quality assessment are provided in Table S2.

### 2.4 Data synthesis and statistics

In this study, we report estimates for the bivariate and hierarchical receiver operating characteristic (HSROC) models (Reitsma et al., 2005).

The bivariate model (Nila et al., 2022) is a statistical approach used in meta‐analysis for diagnostic accuracy studies. It jointly analyses the sensitivity and specificity, allowing for the correlation between these two measures to be accounted for across different studies. This enables a more nuanced understanding of how a particular diagnostic test performs in various settings, providing a summary of both sensitivity and specificity. Specifically, several critical metrics are considered in this model. Logit‐transformed sensitivity reflects the logarithmic transformation of sensitivity values, with the provided mean and confidence interval indicating the precision of this measure. Logit‐transformed sensitivity variance assesses the variability associated with logit‐transformed sensitivity, offering insights into the uncertainty of sensitivity estimates. Logit‐transformed specificity provides a similar analysis but for specificity values. The correlation between sensitivity and specificity quantifies the degree of association between these two critical metrics across studies, shedding light on their interdependence. Lastly, the AUC and partial AUC metrics evaluate the overall diagnostic performance of the test, with partial AUC specifically focusing on a defined range of false positive rates (FPRs), offering valuable information on its discriminative ability within that specific FPR range.

On the other hand, the HSROC model (Trikalinos et al., 2012) offers a more comprehensive view of diagnostic accuracy. It considers both the between‐study variability and the threshold effect, which refers to variations in sensitivity and specificity due to different cut‐off points or thresholds used in various studies. The HSROC model is beneficial in summarizing the overall diagnostic accuracy by considering these variations, providing a summarized ROC curve that includes the different threshold points. In particular, lambda (Λ) captures sensitivity and specificity variations due to different test thresholds used across studies. Theta (Θ) characterizes how sensitivity and specificity change with varying thresholds. Beta (β) quantifies the relationship between the diagnostic odds ratio (DOR) and the threshold. Variance Λ and variance Θ reflect the uncertainties in estimating Λ and Θ. These parameters collectively provide insights into how threshold variations impact diagnostic accuracy and the shape of the receiver operating characteristic curve, enhancing our understanding of a test or biomarker's performance across diverse studies. Overall, the HSROC represents a more sophisticated approach to combining information from various studies, offering a global summary of the diagnostic ability of a particular test or biomarker.

Begg's rank correlation (Begg & Mazumdar, 1994), Egger's (Egger et al., 1997) and Deek's (Deeks et al., 2005) regression tests, funnel plots, andthe trim‐and‐fill method (Shi & Lin, 2019) were used to evaluate publication bias (Lin & Chu, 2018). Funnel plots, a graphical representation showcasing the effect sizes against their precision, serve as a primary visual tool for detecting asymmetry that may hint at publication bias. Begg's rank correlation (Begg & Mazumdar, 1994) assesses bias by determining the correlation between the effect sizes and their respective variances; a significant correlation suggests the presence of bias. Egger's regression test (Egger et al., 1997) evaluates funnel plot asymmetry by regressing the standardized effect sizes against their precision, with a non‐zero intercept signifying potential small‐study effects or biases. Deek's regression test (Deeks et al., 2005) tailored for diagnostic meta‐analyses, contrasts the diagnostic odds ratio with the inverse of the effective sample size's square root; a significant outcome indicates potential publication bias. Lastly, the trim‐and‐fill method (Shi & Lin, 2019) operates on the assumption of a symmetric funnel plot in the absence of bias. This method ‘trims’ the asymmetrical studies and ‘fills’ with estimated missing ones, providing an adjusted effect size.

Finally, if there were more than two ROC models available within the same study, we selected the one with the highest AUC – indicating the most effective biomarker performance in distinguishing patients with parkinsonian disorders among each other or from HCs – for inclusion in the meta‐analysis.

## 3 RESULTS

**FIGURE 1:**
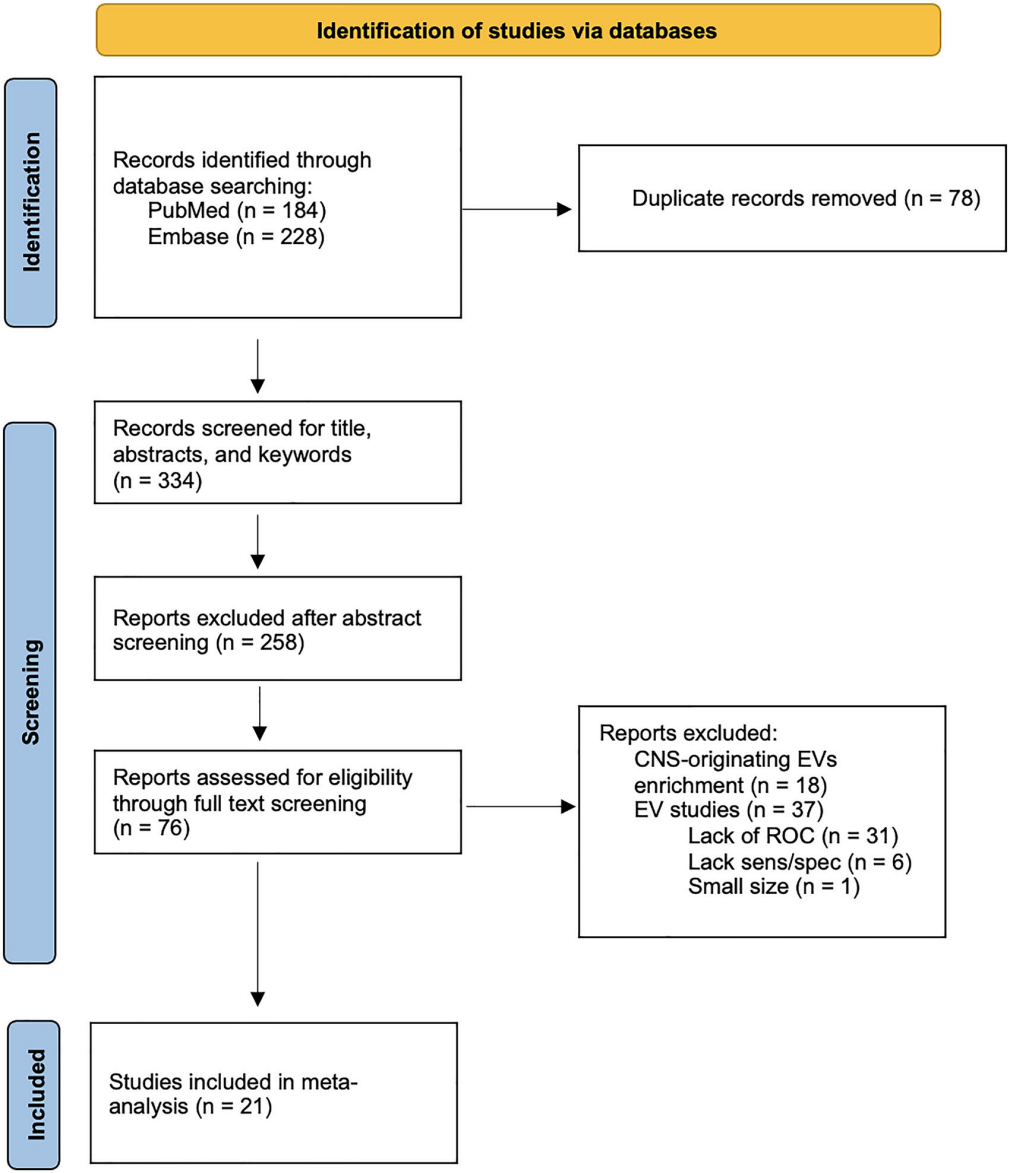
PRISMA flow diagram for inclusion of selected studies.

The systematic and hand search identified 412 articles of which 78 duplicated articles in PUBMED and EMBASE were removed. After title and abstract screening of 334 articles, 76 articles were considered potentially eligible (Figure 1). Following a full‐text screening, 37 studies using general EVs and 18 using only CNS‐originating EVs (Agliardi et al., 2021; Blommer et al., 2023; Dutta et al., 2023; Dutta et al., 2023; Jiang et al., 2020; Jiang et al., 2021; Jiao et al., 2023; Kluge et al., 2022; Meloni et al., 2023; Meloni et al., 2023; Niu et al., 2020; Rani et al., 2019; Shi et al., 2014; Si et al., 2019; Taha et al., 2023; Yu et al., 2020; Zhao et al., 2019; Zou et al., 2020) were excluded. One study used both CNS‐originating EVs and general EVs for ROC analysis, and we did not exclude it (Yan et al., 2022). Among the studies using general EVs, thirty of those studies did not include an ROC analysis (Athauda et al., 2019; Bhattacharyya et al., 2022; Cerri et al., 2018; Chan et al., 2023; Chan et al., 2021; Chan et al., 2023; Chung et al., 2020; Elkouris et al., 2019; Fraser et al., 2016; Han et al., 2019; Herman et al., 2023; Ho et al., 2014; Hong et al., 2023; Kitamura et al., 2018; Lamontagne‐Proulx et al., 2019; Leng et al., 2020; Leng et al., 2022; Miyamoto et al., 2023; Sproviero et al., 2021; Sproviero et al., 2022; Taymans et al., 2023; Wang et al., 2017; Wang et al., 2018; Wang et al., 2020; Wang et al., 2022; Xia et al., 2019; Yang et al., 2015; Yang et al., 2018; Yeh et al., 2021; Zhao et al., 2020), six did not include sensitivity or specificity (Grossi et al., 2021; Gualerzi et al., 2019; He et al., 2021; Ohmichi et al., 2019; Ruf et al., 2021; Yao et al., 2018), and one study only performed the ROC analysis directly in serum instead of EVs (Citterio et al., 2023). In total, the meta‐analysis included 21 studies (Barbagallo et al., 2020; Cao et al., 2019; Cao et al., 2020; Cao et al., 2017; Chung et al., 2021a; Chung et al., 2021b; Dos Santos et al., 2018; Gui et al., 2015; Hadisurya et al., 2023; Hong et al., 2021; Lucien et al., 2022; Manna et al., 2021; Shim et al., 2021; Stuendl et al., 2016; Stuendl et al., 2021; Tong et al., 2022; Vacchi et al., 2020; Wang et al., 2019; Xie et al., 2022; Yan et al., 2022; Zheng et al., 2021) encompassing 1285 patients with PD, 24 with MSA, 105 with DLB, 99 with PSP, 0 with CBS, 101 with RBD, and 783 HCs.

Descriptive characteristics are summarized in Table 1. In one study (Gui et al., 2015), the highest AUC was obtained for a model compromising miR‐153 and miR‐409‐3p (AUC = 0.990). However, the authors did not include the sensitivity and specificity for the combined miR‐153 and miR‐409‐3p model, precluding its inclusion in the meta‐analysis. As such, we discussed the next best model, which was miR‐409‐3p only (AUC = 0.970). Another study (Fraser et al., 2016) was omitted from the meta‐analysis but is included in the systematic review tables due to the small number of participants (*n* = 14).

**TABLE 1:** Demographics and characteristics of the included studies for EV biomarkers.

Study (first author, year) (Country)	EV isolation method	EV confirmation method	Quantification method	# PD	# MSA	# DLB	# PSP	# CBS	# RBD	# HC	Age (years)	Female (%)	Disease Duration (years)	HY scale	UPDRS III	MMSE	MoCA
**CSF**																	
Gui et al. 2015, China	3000×*g* for 15 min followed by 10,000×*g* for 30 min followed by 50,000×*g* for 1 h followed by resuspension in 0.32 M sucrose and 100,000×*g* for 1 h	FC TEM	TaqMan qRT‐PCR	47	0	0	0	0	0	27	PD: 63.0 ± 9.0 HC: 60.0 ± 13.0	PD: 46.8% HC: 33.3%	PD: 11.5 ± 6.6	NA	NA	NA	NA
Stuendl et al. 2016, Germany	3500×*g* for 10 min, followed by 2×4500×*g* for 10 min, followed by 10,000×*g* for 30 min and 100,00×*g* for 60 min	WB EM NTA	ECLIA (Meso Scale Discovery)	37	0	35	25	0	0	0	PD: 73 ± 7.9 DLB: 72 ± 6.85 PSP: 70 ± 7 PNP: 67 ± 14	PD: 70% DLB: 40% PSP: 80% PNP: 40%	PD: 6.42 ± 5.92 DLB: 5 ± 6.67 PSP: 4.42 ± 3 PNP: 5.75 ± 6.58	NA	NA	PD: NA DLB: 18 ± 6.73 PSP: NA PNP: NA	NA
Dos Santos et al. 2018, Belgium	miRCURY Exosome Isolation kit (Exiqon)	NA	qRT‐PCR ELISA (Analytik‐Jena) ECLIA (Meso Scale Discovery) Magnetic Bead (Millipore)	40	0	0	0	0	0	40	Early PD: 61.0 ± 1.0 HC: 64.0 ± 1.0	Early PD: 50% HC: 50%	Early PD: 1.8 ± 1.0	Early PD: 2.0 ± NA	Early PD: 21.0 ± NA	NA	NA
Hong et al. 2021, United States	Apogee nanoscale flow cytometry	FC NTA	Luminex Apogee assay	170 Early (duration < 5 years): 51	0	0	0	0	0	131	PD: 64.5 ± 9.3 Early PD: NA HC: 68.1 ± 10.3	PD: 26.5% Early PD: NA HC: 46.6%	PD: 8.1 ± 5.1 Early PD: NA	NA	PD: 24.8 ± 13.5 Early PD: NA	NA	PD: 24.7 ± 4.0 Early PD: NA
Tong et al. 2022, China	ExoQuick‐TC (Systems Biosciences)	FC	TaqMan qRT‐PCR	209	0	0	0	0	0	50	PD: 68.2 ± 5.4 HC: 65.6 ± 4.3	PD: 45.0% HC: 42.0%	NA	NA	NA	NA	NA
**Plasma**																	
Vacchi et al. 2020, Italy	MACSPlex Human Exosome Kit and FC	WB FC	MACSPlex Human Exosome Kit	27	8	0	0	0	0	19	PD: 66.0 ± 11.8 MSA: 68.0 ± 8.6 HC: 61.0 ± 8.2	PD: 37.0% MSA: 75.0% HC: 47.4%	PD: 4.0 ± NA MSA: 5.5 ± NA	PD: 2.0 ± NA MSA: 5.0 ± NA	PD: 23.0 ± NA MSA: 42.5 ± NA	PD: 30.0 ± NA MSA: 26.0 ± NA	PD: 27.0 ± NA MSA: 24.5 ± NA
Chung et al. 2021a, Taiwan	exoEasy Maxi Kit (Qiagen)	NTA TEM WB	Immunomagnetic Reduction Assay	116	0	0	0	0	0	46	PD: 69.7 ± 8.4 HC: 67.0 ± 7.0	PD: 46.5% HC: 60.9%	PD: 2.8 ± 2.5	NA	PD (UPDRS II): 7.9 ± 5.8	PD: 24.2 ± 6.4	NA
Chung et al. 2021b, Taiwan	exoEasy Maxi Kit (Qiagen)	NTA TEM WB	Immunomagnetic Reduction Assay	116	0	0	0	0	0	46	PD: 69.7 ± 8.4 HC: 67.0 ± 7.0	PD: 46.5% HC: 60.9%	PD: 2.8 ± 2.5	NA	PD (UPDRS II): 7.9 ± 5.8	PD: 24.2 ± 6.4	PD: 20.4 ± 6.0
Shim et al. 2021, South Korea	20,000×*g* for 1h followed by 100,000×*g* for 1.5 h	NTA WB TEM	Enzyme Activity Assay (Abcam)	34	0	0	0	0	0	29	PD: 74.2 ± 4.7 HC: 73.9 ± 4.6	PD: 11.8% HC: 17.2%	PD: 5.1 ± 4.3	PD: 1.5 ± 0.56	PD: 40.0 ± 13.1	PD: 24.7 ± 3.8	NA
Zheng et al. 2021, China	Total Exosome Isolation kit (Invitrogen)	NTA TEM WB	WB	36	0	0	0	0	0	36	PD: 70.4 ± 0.6 HC: 69.2 ± 0.3	PD: 47.2% HC: 52.8%	NA	NA	NA	NA	NA
Stuendl et al. 2021, Germany	SEC	NTA WB TEM	ECLIA	96	0	50	50	0	0	50	PD: 65.0 + 11.8 DLB: 70.8 ± 6.5 PSP: 69.4 ± 5.7 HC: 61.6 ± 14.1	PD: 46.3% DLB: 32.0% PSP: 66.0% HC: 55.5%	PD: 7.6 ± 5.6 DLB: 2.6 ± 2.0 PSP: 3.6 ± 3.0	PD: 2.2 ± 0.7 DLB: NA PSP: NA	NA	PD: 27.8 ± 2.5 DLB: 18.2 ± 5.7 PSP: NA	NA
Lucien et al. 2022, United States	Apogee Nanoscale Flow Cytometry	WB	WB	20	0	20	0	0	0	20	PD: NA HC: NA	PD: 30% HC: NA	NA	PD: 1 ± NA	PD: 15 ± NA	NA	NA
Xie et al. 2022, China	exoEasy Max Kit (Qiagen)	DLS TEM WB	qRT‐PCR WB	30	0	0	0	0	0	30	PD: 60.0 ± 7.9 HC: 58.2 ± 9.4	PD: 43.3% HC: 43.3%	NA	NA	NA	NA	NA
Yan et al. 2022	ExoQuick (Systems Biosciences)	NTA TEM WB	WB	44 Early: 28 Advanced: 16	0	0	0	0	101	48	PD: 64.2 ± 9.6 Early: 63.2 ± 9.8 Advanced: 65.9 ± 9.2 RBD: 61.9 ± 7.9 HC: 61.5 ± 7.1	PD: 56.8% Early: 53.6% Advanced: 62.5% RBD: 56.4% HC: 54.2%	PD: 3.7 ± 3.8 Early: 2.5 ± 3.0 Advanced: 5.7 ± 4.1 RBD: NA	PD:2.1 ± 1.0 Early: 1.6 ± 0.5 Advanced: 3.1 ± 0.8 RBD: NA	PD: 32.0 ± 19.7 Early: 21.8 ± 10.7 Advanced: 49.7 ± 19.5 RBD: NA	PD: 23.7 ± 6.2 Early: 25.3 ± 4.8 Advanced: 21.1 ± 7.4 RBD: 21.8 ± 5.9	PD: 19.2 ± 7.1 Early: 21.0 ± 5.8 Advanced: 15.9 ± 8.2 RBD: 16.6 ± 6.4
**Serum**																	
Cao et al. 2017, China	2000×*g* for 30 min followed by Total Exosome Isolation Reagent (Thermo Fisher Scientific)	Exosome‐Human CD63 Detection Reagent FC	qRT‐PCR	109	0	0	0	0	0	40	PD: 69.8 ± 6.2 HC: 67.9 ± 8.6	PD: 33.0% HC: 37.5%	PD: 9.2 ± 7.4	NA	NA	NA	NA
Barbagallo et al. 2020, Italy	ExoQuick (Systems Biosciences)	None (quality low)	TaqMan RT‐PCR	PD: 30 VP: 25	0	0	0	0	0	30	PD: 69.6 ± 8.0 HC: 67.9 ± 8.2 VP: 74.9 ± 6.6	PD: 20.1% HC: 66.7% VP: 36.0%	PD: 6.9 ± 3.6 VP: 4.7 ± 3.0	PD: 2.6 + 0.9 VP: 2.6 ± 0.7	PD: 36.9 ± 14.4 VP: 35.5 ± 13.3	PD: 25.9 ± 3.5 VP: 24.9 ± 3.8	NA
Manna et al. 2021, Italy	3000×*g* for 10 min followed by ExoQuick (Systems Biosciences	None (quality low)	TaqMan RT‐qPCR	40	0	0	24	0	0	39	PD: 66.4 ± 8.6 PSP: 71.5 +± 5.4 HC: 63.7 ± 7.5	PD: 42.2% PSP: 37.5% HC: 61.5%	PD: 5.6 ± 4.1 PSP: 4.4 ± 2.4	PD: 2.0 ± NA PSP: 4.0 ± NA	PD: 24.0 ± NA PSP: 47.0 ± NA	NA	NA
Tong et al. 2022, China	ExoQuick‐TC (Systems Biosciences)	FC	TaqMan qRT‐PCR	209	0	0	0	0	0	50	PD: 68.2 ± 5.4 HC: 65.6 ± 4.3	PD: 45.0% HC: 42.0%	NA	NA	NA	NA	NA
**Urine**																	
Fraser et al. 2016, United States	500×*g* for 10 min followed by 10,000×*g* for 30 min followed by 100,000×*g* followed by resuspension into 1 g/mL iodixanol followed by 50,000×*g* for 16 h	CEM WB	WB	PD: 14 LRRK2+ PD: 7	0	0	0	0	0	3	PD: 69 ± NA LRRK2+ PD: 63 ± NA HC: 67 ± NA	PD: 0% LRRK2+ PD: 0% HC: 0%	NA	NA	PD: 12 ± NA LRRK2+ PD: 22 ± NA	NA	PD: 27 ± NA LRRK2 PD: 20.5 ± NA
Wang et al. 2019, United States.	10,000×*g* for 30 min followed by 2×100,000×*g* for 1 h at 4°C	NTA CryoEM	Immunoblot	52	0	0	0	0	0	56	PD: 65.0 ± NA HC: 62.5 ± NA	PD: 44.2% HC: 51.8%	PD: 7.0 ± NA	NA	PD: 26.0 ± NA HC: 0 ± NA	NA	PD: 25.0 ± NA HC: 27 ± NA
Hadisurya et al. 2023, United States.	EVTrap (Tymora Analytical operations)	WB	LC‐MS	48 (including LRRK2+ PD).	0	0	0	0	0	32	PD: 66.1 ± NA HC: 69.4 ± NA LRRK2 PD: 69.7 ± NA	PD: 42.9% HC: 47.6% LRRK2 PD: 45%	PD: 0–18 (range) LRRK2 PD: 1–26 (range)	NA	PD: 17.5 ± NA LRRK2 PD: 21.1 ± NA	NA	PD: 27.2 ± NA LRRK2 PD: 26.2 ± NA
**Saliva**																	
Cao et al. 2019, China.	XYCQ EV Enrichment Kit	NTA TEM WB IP	ECLIA	74	0	0	0	0	0	60	PD: 59.6 ± 8.6 HC: 58.8 ± 9.8	PD: 45.9% HC: 56.7%	PD: 5.5 ± NA	PD: 2.5 ± NA	PD: 38.4 ± 19.4	NA	NA
Cao et al. 2020, China.	XYCQ EV Enrichment Kit	NTA TEM WB	ECLIA	26	16	0	0	0	0	0	PD: 56.8 ± 6.4 MSA: 57.3 ± 7.8	PD: 53.8% MSA: 43.7%	PD: 2.6 ± 1.2 MSA: 3.1 ± 1.7	PD: 2.5 ± 0.6 MSA: 2.8 ± 0.7	PD: 40.8 ± 16.0 MSA: 42.7 ± 18.9	NA	NA

The majority of the studies analysed were found to be of high quality, as outlined in Table S3. Nevertheless, there was an absence of transparent reporting regarding the method of sampling, obscuring the ability to properly evaluate the risk of bias in the selection of patients, which we deemed as unclear. One study was deemed as high risk of bias (Wang et al., 2019) due to the exclusion of four participants who had undetectable levels of Calbindin. In the area of biomarker measurement utilizing EVs, the risk of bias was deemed low since this objective measure is not influenced by prior information about the patient's clinical condition. Regarding the flow and timing domain, the risk of bias was considered low across all studies, as the interval between clinical diagnosis and biomarker measurement could be estimated. Although the bulk of the articles (76.2%) were judged to have a low risk of bias in the reference standard domain, five studies (Lucien et al., 2022; Manna et al., 2021; Wang et al., 2019; Yan et al., 2022; Zheng et al., 2021) were identified as having a high risk of bias due to the lack of quantification using highly sensitive methods in comparison to a reference standard.

We attempted to conduct a comprehensive meta‐analysis, evaluating the diagnostic accuracy of biomarkers in EVs for parkinsonian disorders against one another and against HCs. However, as the number of studies in all other scenarios was ≤ 3, it precluded us from conducting meaningful analyses. As such, we only conducted a meta‐analysis for patients with PD versus HCs, but also included relevant descriptive statistics for other scenarios in Table 2.

**TABLE 2:** Descriptive statistics of the diagnostic metrics of studies included in the meta‐analysis.

Study	Biomarkers	N (in analysis)	Sensitivity (95% CI)	Specificity (95% CI)	FPR (95% CI)	DOR (95% CI)	posLR (95% CI)	negLR (95% CI)
**PD versus Control**								
Gui et al. 2015	miR‐409‐3p	74	0.90 (0.81–0.95)	0.90 (0.81–0.95)	0.10 (0.05–0.19)	81.00 (27.87–235.41)	9.00 (4.55–17.82)	0.11 (0.06–0.22)
Hong et al. 2021	Aggregated α‐synuclein/total α‐synuclein	301	0.97 (0.91–0.99)	0.90 (0.81–0.94)	0.10 (0.06–0.19)	267.82 (63.09–1137.02)	9.23 (4.88–17.47)	0.03 (0.01–0.12)
Tong et al. 2022	miR‐214	259	0.80 (0.75–0.84)	0.71 (0.66–0.76)	0.29 (0.24–0.34)	9.79 (6.72–14.25)	2.76 (2.29–3.32)	0.28 (0.22–0.36)
Vacchi et al. 2020	CD2, CD41b, MCSP	46	0.62 (0.49–0.73)	0.79 (0.67–0.87)	0.21 (0.13–0.33)	6.03 (2.75–13.21)	2.93 (1.75–4.88)	0.49 (0.35–0.68)
Chung et al. 2021a	Age, sex, alpha‐synuclein, AB1‐42 and tau	162	0.82 (0.77–0.86)	0.82 (0.77–0.86)	0.18 (0.14–0.23)	21.08 (13.46–33.01)	4.59 (3.52–5.99)	0.22 (0.17–0.28)
Chung et al. 2021b	α‐synuclein	162	0.50 (0.42–0.58)	0.76 (0.69–0.82)	0.24 (0.18–0.31)	3.13 (1.95–5.01)	2.06 (1.51–2.82)	0.66 (0.55–0.79)
Shim et al. 2021	AChE activity	63	1.00 (0.97–1.00)	0.60 (0.52–0.67)	0.40 (0.33–0.48)	483.78 (29.61–7905.39)	2.48 (2.06–2.99)	0.01 (0.00–0.08)
Zheng et al. 2021	Oligomeric α‐synuclein/total α‐synuclein	72	0.83 (0.76–0.88)	0.60 (0.52–0.68)	0.40 (0.32–0.48)	7.56 (4.39–13.05)	2.09 (1.70–2.59)	0.28 (0.19–0.41)
Zheng et al. 2021	Phosphorylated oligomeric α‐synuclein/total phosphorylated α‐synuclein	72	0.61 (0.49–0.71)	0.60 (0.48–0.70)	0.40 (0.30–0.52)	2.30 (1.19–4.47)	1.51 (1.08–2.11)	0.66 (0.47–0.92)
Stuendl et al. 2021	α‐synuclein/EV particle concentration	146	0.80 (0.66–0.89)	0.88 (0.76–0.95)	0.12 (0.05–0.24)	29.79 (9.53–93.06)	6.82 (3.07–15.15)	0.23 (0.13–0.41)
Lucien et al. 2022	α‐synuclein positive EVs	40	0.91 (0.79–0.97)	0.79 (0.65–0.89)	0.21 (0.11–0.35)	40.97 (10.83–154.97)	4.41 (2.41–8.09)	0.11 (0.04–0.30)
Xie et al. 2022	hsa‐miR‐15b‐5p hsa‐miR‐30c‐2‐3p hsa‐miR‐138‐5p hsa‐miR‐106b‐3p	60	0.60 (0.48–0.70)	0.60 (0.48–0.70)	0.40 (0.30–0.52)	2.17 (1.12–4.21)	1.48 (1.05–2.07)	0.68 (0.48–0.95)
Yan et al. 2022	α‐synuclein	92	0.93 (0.83–0.97)	0.76 (0.64–0.85)	0.24 (0.15–0.36)	40.26 (13.05–124.22)	3.90 (2.47–6.14)	0.10 (0.04–0.24)
Cao et al. 2017	miR‐195 miR19b miR‐24	149	0.85 (0.78–0.90)	0.90 (0.84–0.94)	0.10 (0.06–0.16)	49.17 (24.64–98.12)	8.23 (5.11–13.24)	0.17 (0.11–0.25)
Barbagallo et al. 2020	Let‐7d miR‐22*, miR‐23a miR‐24 miR‐142‐3p miR‐222	60	0.88 (0.77–0.94)	0.86 (0.75–0.93)	0.14 (0.07–0.25)	44.06 (15.37–126.30)	6.29 (3.35–11.83)	0.14 (0.07–0.28)
Manna et al. 2021	miR‐21‐3p miR‐22‐3p miR‐223‐5p	73	0.72 (0.61–0.81)	0.78 (0.67–0.86)	0.22 (0.14–0.33)	9.10 (4.31–19.21)	3.24 (2.07–5.08)	0.36 (0.24–0.53)
Manna et al. 2021	miR‐21‐3p miR‐22‐3p miR‐199a‐5p miR‐223‐5p miR‐425‐5p Let‐7i‐5p miR‐483‐5p miR‐29a‐3p	73	0.57 (0.46–0.68)	0.99 (0.94–1.00)	0.01 (0.00–0.06)	198.33 (11.83–3324.53)	85.00 (5.33–1355.65)	0.43 (0.33–0.56)
Tong et al. 2022	miR‐214	259	0.71 (0.62–0.79)	0.69 (0.60–0.77)	0.31 (0.23–0.40)	5.54 (3.10–9.91)	2.31 (1.70–3.14)	0.42 (0.30–0.58)
Wang et al. 2019	Calbindin and SNAP23	108	0.98 (0.84–1.00)	0.67 (0.48–0.82)	0.33 (0.18–0.52)	105.00 (5.68–1939.71)	3.00 (1.72–5.22)	0.03 (0.00–0.45)
Hadisurya et al. 2023	HNRNPA1 PCSK1N pLTB4R pLA2G4A pPPFIA1 pRRR15	25	0.91 (0.86–0.95)	0.86 (0.79–0.91)	0.14 (0.089–0.21)	63.61 (29.40–137.60)	6.33 (4.19–9.58)	0.10 (0.06–0.17)
Cao et al. 2019	Oligomeric α‐synuclein	134	0.81 (0.74–0.87)	0.71 (0.63–0.78)	0.29 (0.22–0.37)	10.38 (5.88–18.33)	2.77 (2.11–3.65)	0.27 (0.19–0.38)
Cao et al. 2019	Oligomeric α‐synuclein/total α‐synuclein	134	0.92 (0.80–0.97)	0.64 (0.49–0.77)	0.36 (0.23–0.51)	20.02 (5.69–70.44)	2.55 (1.69–3.83)	0.13 (0.039–0.36)
**Early‐stage PD versus Control**								
Dos Santos et al. 2018	miR‐10b‐5p miR‐151a‐3p miR‐22‐3p α‐syn.	80	0.98 (0.91–0.99)	0.78 (0.67–0.85)	0.23 (0.15–0.33)	134.33 (30.02–601.11)	4.33 (2.88–6.52)	0.03 (0.099–0.13)
Hong et al. 2021	Aggregated α‐synuclein/total α‐synuclein	182	0.90 (0.85–0.94)	0.73 (0.66–0.79)	0.27 (0.21–0.34)	24.73 (13.76–44.46)	3.35 (2.62–4.27)	0.14 (0.089–0.21)
**PD versus MSA**								
Cao et al. 2020	α‐synuclein	42	0.92 (0.81 −0.97)	0.64 (0.49–0.77)	0.36 ( 0.23–0.51)	23.4 (6.17–88.76)	2.6 (1.72–3.93)	0.11 (0.036–0.34)
**PD versus DLB**								
Stuendl et al. 2016	EV α‐synuclein	72	0.86 (0.76–0.92)	0.78 (0.67–0.86)	0.22 (0.14–0.33)	21.70 (9.10–51.73)	3.88 (2.49–6.03)	0.18 (0.10–0.32)
Stuendl et al. 2021	α‐synuclein/EV particle concentration	146	0.81 (0.74–0.86)	0.73 (0.66–0.80)	0.27 (0.20–0.34)	11.56 (6.66–20.07)	3.03 (2.29–4.00)	0.26 (0.19–0.37)
**PD versus PSP**								
Stuendl et al. 2016	EV α‐synuclein/total EV concentration	62	0.85 (0.97–0.97)	0.79 (0.67–0.87)	0.21 (0.13–0.33)	54.65 (16.74–178.47)	4.46 (2.74–7.27)	0.079 (0.03–0.21)
Stuendl et al. 2021	α‐synuclein/EV particle concentration	146	0.74 (0.87–0.87)	0.69 (0.61–0.76)	0.31 (0.24–0.39)	9.89 (5.73–17.08)	2.64 (2.05–3.41)	0.27 (0.19–0.38)
Manna et al. 2021	miR‐425‐p miR‐21‐3p miR‐199a‐5p	60	0.72 (0.91–0.91)	0.85 (0.74–0.92)	0.15 (0.08–0.26)	28.33 (10.62–75.60)	5.56 (3.01–10.25)	0.20 (0.11–0.35)
Manna et al. 2021	miR‐425‐5p miR‐21‐3p miR‐199a‐5p miR‐483‐5p miR‐22‐3p miR‐29a‐3p	60	0.78 (0.94–0.94)	0.90 (0.80–0.95)	0.10 (0.05–0.20)	68.14 (21.48–216.16)	8.83 (4.11–18.98)	0.13 (0.06–0.26)
**MSA versus Control**								
Vacchi et al. 2020	CD2 CD62P CD146	27	0.982 (0.85–0.99)	0.839 (0.664–0.931)	0.16 (0.067–0.34)	(287.22 (14.69–5616.28	6.11 (2.62–14.27)	0.021 (0.001–0.33)
**PSP versus Control**								
Manna et al. 2021	miR‐22‐3p miR‐425‐5p	53	0.72 (0.61–0.81)	0.78 (0.67–0.86)	9.10 (4.31–19.21)	3.24 (2.07–5.08)	0.36 (0.24–0.53)	0.23 (0.14–0.33)
Manna et al. 2021	miR‐483‐5p miR‐22‐3p miR‐425‐5p miR‐21‐3p miR‐223‐5p miR‐29a‐3p	53	0.57 (0.46–0.68)	0.99 (0.94–1.00)	198.33 (11.83–3324.53)	85.00 (5.33–1355.65)	0.43 (0.33–0.56)	0.00068 (0.00071 – 0.061)
**DLB versus PSP**								
Stuendl et al. 2016	α‐synuclein	60	0.72 (0.59–0.81)	0.92 (0.82–0.96	27.82 (9.05 −81.4)	8.60 (3.66 −20.20)	0.31 (0.20–0.47)	0.083 (0.036–0.18)

### 3.1 Diagnosing Parkinson's disease against healthy controls

**FIGURE 2:**
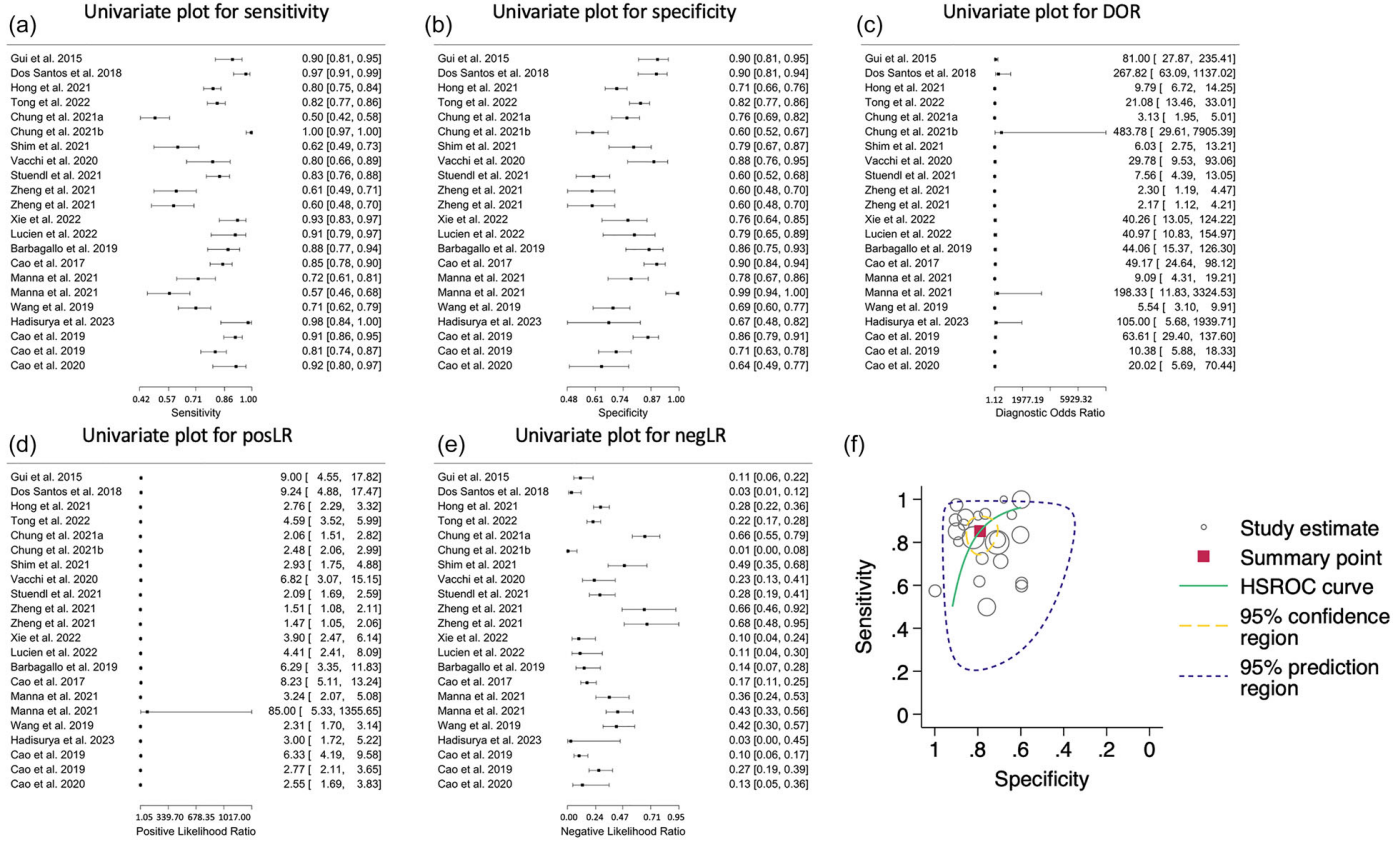
Diagnostic accuracy of biomarkers in extracellular vesicles (EVs) for the differential diagnosis of Parkinson's disease (PD) from healthy controls (HCs). (a‐e) Univariate Forest plots for sensitivity, specificity, diagnostic odds ratio (DOR), positive (posLR) and negative (negLR) likelihood ratios, respectively. (f) Summary receiver operating characteristics (SROC). The dotted circle shows the mean summary estimate of sensitivities and specificities using a bivariate model. The summary line is obtained from a hierarchical SROC model.

In this meta‐analysis, we employed a bivariate and HSROC model to measure the diagnostic test's accuracy for patients with PD versus HCs. Descriptive statistics including sensitivity, specificity, FPR, DOR, positive likelihood ratio (posLR), and negative likelihood ratio (negLR) for each individual study are summarized in Table 2. Pooled summary of sensitivity (Figure 2a), specificity (Figure 2b), DOR (Figure 2c), posLR (Figure 2d) and negLR (Figure 2e), and the bivariate and HSROC models’ (Figure 2f) statistics are summarized in Table 3.

**TABLE 3:** Meta‐analysis of diagnostic accuracy for patients with Parkinson's disease (PD) versus healthy controls (HCs) summary statistics for the bivariate and hierarchal summary receiver operating characteristic (HSROC) models.

Model	Variable	Coefficient estimate ± SE (95% CI)
**Summary statistic**		
	Sensitivity	0.85 ± 0.03 (0.78–0.91)
	Specificity	0.79 ± 0.03 (0.73–0.84)
	DOR	21.58 ± 6.50 (11.96–38.95)
	posLR	4.04 ± 0.54 (3.10–5.26)
	negLR	0.19 ± 0.042 (0.12–0.29)
	1/negLR	5.34 ± 1.20 (3.44–8.28)
**Bivariate**		
	Logit‐transformed sensitivity	1.75 ± 0.26 (1.23–2.27)
	Logit‐transformed sensitivity variance	1.32 ± 0.51 (0.61–2.82)
	Logit‐transformed specificity	1.32 ± 0.17 (0.99–1.64)
	Logit‐transformed specificity variance	0.52 ± 0.20 (0.24–1.12)
	Correlation between sensitivity and specificity	‐0.080 ± 0.24 (−0.050–0.37)
	AUC (partial AUC)	0.852 (0.672)
**HSROC**		
	Lambda (Λ)	3.05 ± 0.29 (2.49–3.62)
	Theta (Θ)	‐0.14 ± 0.24 (−0.62–0.34)
	Beta (β)	‐0.47 ± 0.28 (−1.01–0.074)
	Variance Λ	0.15 ± 0.54 (0.75–3.03)
	Variance Θ	0.44 ± 0.17 (0.22–0.93)

The bivariate model revealed a moderate diagnostic sensitivity (85.2%, 95% CI: 0.77–0.91) and specificity (78.9%, 95% CI: 0.73–0.84) for patients with PD versus HCs. Though the variance in sensitivity (Figure 2a) and specificity (Figure 2b) across the 21 studies indicated substantial heterogeneity, as seen graphically and by the Chi‐square (*χ* ^2^) test for equality of sensitivities (χ2 = 264.22, *p* < 0.0001) and specificities (*χ* ^2^ = 150.81, *p*‐value < 0.0001). This heterogeneity could arise from the difference in the isolation, quantification methodologies, populations included, or the medium used for EV isolation in each study. Furthermore, the correlation between the logit transformations of sensitivity and specificity using a bivariate model was found to be negligible (*r* = ‐0.079, 95% CI: ‐0.50–0.37), suggesting that these two measures operate relatively independently. Lastly, the pooled AUC was 0.852 and the partial AUC, focusing on a specific range of FPRs, was 0.672, indicating fair diagnostic accuracy.

In the second part of our analysis, we utilized the HSROC model. Here, the measure of overall test accuracy, denoted by lambda is 3.05, translates to approximately a DOR of 21.2 (95% CI: 12.0–37.5), indicating good accuracy in distinguishing between patients with PD versus HCs. The theta value was approximately ‐0.14, which suggests that the test performs consistently across different diagnostic thresholds. The asymmetry of the ROC curve, denoted by the beta value of ‐0.47, shows a slight tendency towards a trade‐off between sensitivity and specificity, however, non‐significant (p = 0.091). Notably, the substantial variance in accuracy (σ^2^_α_ = 1.52, 95% CI: 0.75–3.03) and threshold (σ^2^_θ_ = 0.44, 95% CI: 0.22–0.93) across the studies highlights that there is considerable variability in both the test's accuracy and its performance at different thresholds.

While the diagnostic test shows good performance in diagnosing patients with PD versus HCs, as evidenced by the sensitivity, specificity, and AUC values from the bivariate model, and the overall good accuracy from the HSROC model (Figure 2f), the substantial variability and heterogeneity across the studies calls for cautious interpretation of these results and emphasizes the need for further independent validations in diverse settings and populations.

Notably, among the studies, the presumed best biomarker offering a balance between sensitivity and specificity utilizing a large sample size is the one integrating the aggregated α‐synuclein and total α‐synuclein (Hong et al., 2021). This finding aligns with the mechanistic understanding that in PD when α‐synuclein misfolds, cells may try to release more of it into EVs to reduce its intracellular toxic effects (Hill, 2019). However, discrepancies in methodologies and patient profiles (Table 1) pose challenges in identifying a single superior biomarker. The effectiveness of a biomarker can vary based on its application context, disease stage, or patient demographics, among many other factors, which are not standardized across the included studies.

**FIGURE 3:**
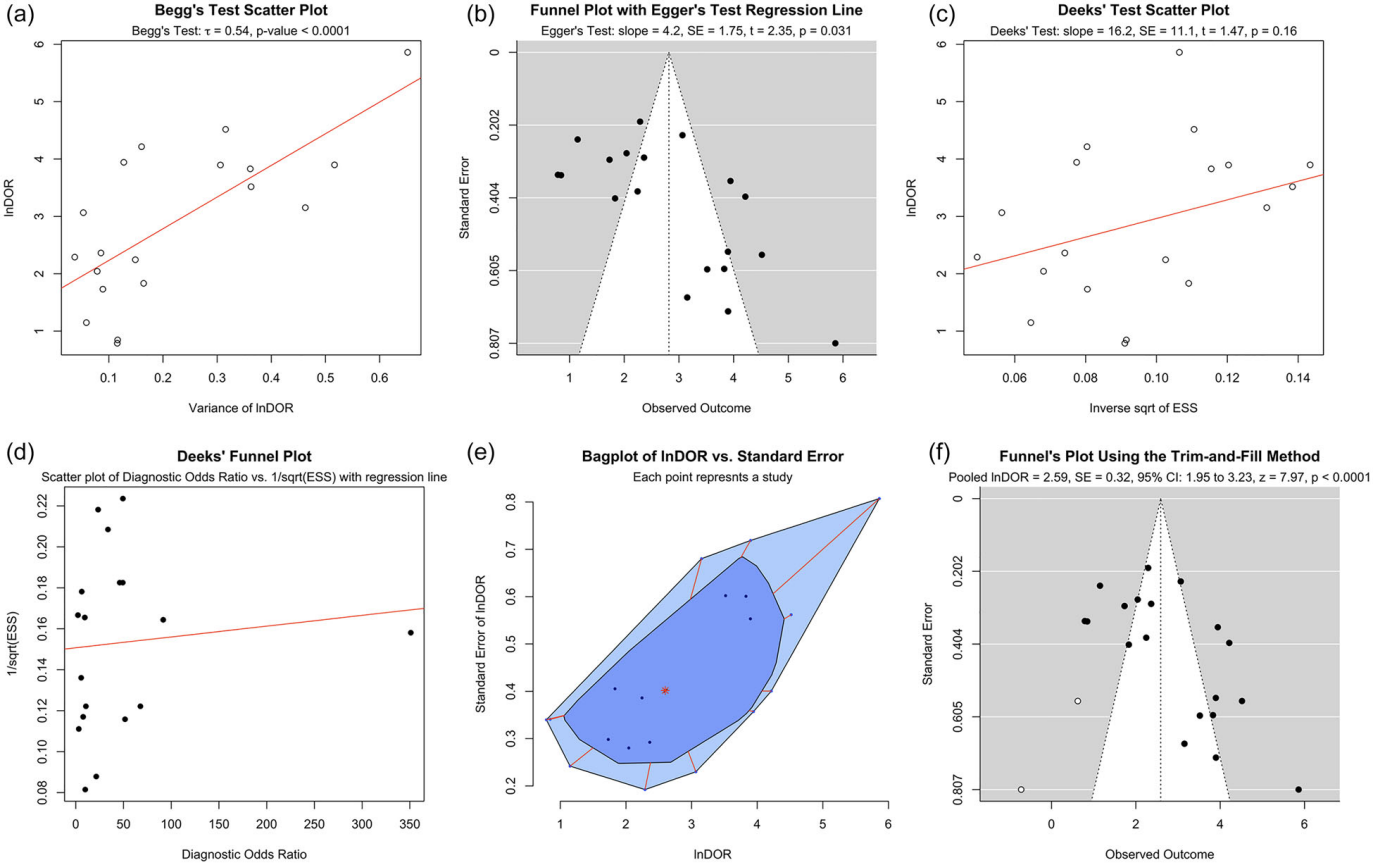
Publication bias was assessed using (a) Begg's correlation, (b) Egger's regression, (c) Deek's regression, (d) Deek's funnel plot, (e) a bagplot and (f) a Funnel plot after application of the trim‐and‐fill method for biomarkers in extracellular vesicles (EVs) for the differential diagnosis of patients with Parkinson's disease (PD) from healthy controls (HCs). Collectively, they suggested a substantial presence of publication bias. The trim‐and‐fill method estimated two missing studies (shown as white circles) on the left side of the figure with either small or null diagnostic accuracy. The dotted circle shows the mean summary estimate of sensitivities and specificities using a bivariate model. The summary line is obtained from a hierarchical SROC (HSROC) model.

We further assessed publication bias using Begg's correlation test (Figure 3a), Egger's test (Figure 3b), Deek's test (Figure 3c), a funnel plot (Figure 3d), a bagplot (Figure 3e) and the trim‐and‐fill method (Figure 3f). All tests revealed substantial publication bias except for Deek's test. The trim‐and‐fill method suggested that the unpublished studies are hypothesized to be on the left side of the funnel plot (white circles in Figure 3f) with null or low DOR. This suggested inflation of the perceived diagnostic efficacy of the test, similar to what is observed with CNS‐originating EVs (Taha & Bogoniewski, 2023), but to a much lower degree.

### 3.2 Diagnosing Parkinson's disease against healthy controls by EVs isolation medium

As the size, purity, content, and reliability of EVs depend on media (e.g., CSF, serum, plasma, etc.) and methodology of isolation (Dhondt et al., 2023; Erdbrugger et al., 2021; Krusic Alic et al., 2022; Taha, 2023), we compared the diagnostic metrics by CSF, plasma, and serum. We did not include urine or saliva due to the inclusion of only two studies for each. In one study, both CSF and serum were used (Tong et al., 2022), and were included in both analyses. Four studies quantified biomarkers in EVs isolated from CSF, nine from plasma and four from serum for the differential diagnosis of patients with PD from HCs. Comparative summary of the models’ statistics is included in Table 4.

**TABLE 4:** Comparison of meta‐analysis of diagnostic accuracy for patients with Parkinson's disease (PD) versus healthy controls (HCs) by medium of isolation. CSF—Cerebrospinal fluid. SE—Standard error. AUC—Area under the curve. HSROC—Hierarchal summary receiver operating characteristics.

		CSF	Plasma	Serum
Model	Variable	Coefficient estimate ± SE (95% CI)		
**Summary statistic**				
	Sensitivity	0.88 ± 0.036 (0.79−0.94)	0.83 ± 0.07 (0.65−0.92)	0.79 ± 0.043 (0.69−0.86)
	Specificity	0.85 ± 0.042 (0.75−0.91)	0.71 ± 0.03 (0.64−0.77)	0.91 ± 0.048 (0.76−0.97)
	DOR	42.36 ± 26.66 (12.34−145.45)	11.55 ± 5.51 (4.53−29.45)	35.63 ± 16.25 (14.57−87.12)
	posLR	5.77 ± 1.76 (3.18−10.50)	2.83 ± 0.36 (2.21−3.63)	8.41 ± 4.09 (3.24−21.82)
	negLR	0.14 ± 0.048 (0.069−0.27)	0.25 ± 0.10 (0.11−0.52)	0.24 ± 0.041 (0.17−0.33)
	1/negLR	7.34 ± 2.58 (3.68−14.61)	4.08 ± 1.59 (1.90−8.74)	4.24 ± 0.74 (3.01−5.98)
**Bivariate**				
	Logit–transformed sensitivity	2.04 ± 0.36 (1.34−2.73)	1.56 ± 0.48 (0.63−2.49)	1.30 ± 0.26 (0.79−1.81)
	Logit‐transformed sensitivity variance	1.71 ± 0.32 (1.07−2.34)	0.89 ± 0.16 (0.58−1.19)	2.27 ± 0.57 (1.15−3.40)
	Logit‐transformed specificity	0.35 ± 0.38 (0.04−2.83)	2.05 ± 1.17 (0.67−6.29)	0.27 ± 0.22 (0.06−1.30)
	Logit‐transformed specificity variance	0.33 ± 0.29 (0.06−1.85)	0.17 ± 0.11 (0.04‐0.64)	1.28 ± 1.38 (0.15‐10.67)
	Correlation between sensitivity and specificity	0.98 ± 0.11 (−0.98−1.00)	‐0.19 ± 0.36 (−0.73−0.49)	‐0.79 ± 0.27 (−0.98−0.30)
	AUC (partial AUC)	0.924 (0.883)	0.772 (0.611)	0.884 (0.544)
**HSROC**				
	Lambda (Λ)	3.74 ± 0.63 (2.51−4.97)	2.49 ± 0.39 (1.73−3.26)	3.46 ± 0.38 (2.71−4.21)
	Theta (Θ)	0.13 ± 0.38 (−0.61−0.87)	‐0.41 ± 0.32 (−1.03−0.21)	0.18 ± 0.55 (−0.90−1.27)
	Beta (β)	‐0.032 ± 0.50 (−1.01−0.95)	‐1.25 ± 0.44 (−2.12−0.38)	0.77 ± 0.58 (−0.37−1.90)
	Variance Λ	1.36 ± 1.13 (0.27−6.96)	0.95 ± 0.59 (0.28−3.20)	0.25 ± 0.30 (0.02−2.67)
	Variance Θ	0.0039 ± 0.019 (3.4e^‐6^−45.37)	0.35 ± 0.19 (0.12−1.03)	0.53 ± 0.43 (0.11−2.63)

CSF is widely used for the discovery of biomarkers for neurodegenerative conditions, including parkinsonian disorders, due to its direct connection with the brain. On the other hand, plasma and serum are often used due to their minimally invasive nature, foregoing the need to undergo a lumbar puncture procedure. Therefore, measurement of biomarkers in EVs isolated from CSF, plasma, and serum has been appealing to different groups based on their goal.

CSF‐EVs analysis of sensitivity (Figure S1A), specificity (Figure S1B), DOR (Figure S1C), posLR (Figure S1D) and negLR (Figure S1E) revealed overall better diagnostic accuracy than the combined model above without evidence for publication bias (Figure S2A‐2F). Plasma‐EVs analysis revealed the lowest diagnostic performance (Figures S3A‐3F), with substantial publication bias, as evidenced by Begg's correlation test (Figure S4A) and Egger's regression test (Figure S4B) but not Deek's test or a funnel plot (Figure S4C‐4E). Though the trim‐and‐fill method suggested publication bias, no missing studies were identified (Figure S4F). On the other hand, serum had a moderate diagnostic accuracy (Figures S5A‐5F) with no publication bias (Figures S6A‐6F).

**FIGURE 4:**
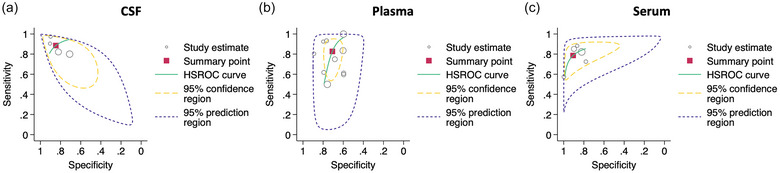
Summary statistics from the bivariate and hierarchical receiver operating characteristics models comparing biomarkers in extracellular vesicles (EVs) isolated from (a) cerebrospinal fluid (CSF), (b) plasma or (c) serum.

Meta‐analyses using the bivariate and HSROC model of CSF, plasma, and serum‐EVs supported the findings, with the highest diagnostic accuracy obtained for CSF (Figure 4a) versus plasma (Figure 4b) and serum (Figure 4c). However, due to the small number of studies using CSF (n = 4), making concise conclusions is difficult. But as mentioned above, due to the CSF's direct connection with the brain, one would expect biomarkers in the CSF to give more of a realistic picture of the brain's biochemistry in comparison to plasma and serum. Nonetheless, in all cases, heterogeneity was large, indicating that further studies are needed to confirm this perceived effect.

### 3.3 Diagnosing Parkinson's disease against healthy controls: General EVs versus CNS‐originating EVs

As EVs are thought to reflect the status of the parent cell by carrying cell‐state‐specific messages (Dixson et al., 2023), many studies have attempted to measure biomarkers in CNS‐originating EVs (Dutta et al., 2023) isolated from the blood. These studies aimed to differentially diagnose patients with PD versus HCs, and other parkinsonian disorders, with the intention of gaining insights into the brain's biochemistry.

Two recent meta‐analyses evaluated the levels of α‐synuclein and biomarker diagnostic accuracy in CNS‐originating EVs and found substantial heterogeneity, publication bias and inconsistency in the findings (Taha & Ati, 2023; Taha & Bogoniewski, 2023). As such, we compared the diagnostic accuracy of biomarkers in general EVs versus CNS‐originating EVs (Taha & Bogonwieski, 2023).

Comparison of the bivariate and HSROC model statistics revealed that biomarkers in general EVs have a higher diagnostic accuracy versus CNS‐originating EVs (Table 5). Though there was substantial publication bias in both methodologies, the trim‐and‐fill method estimated only two missing studies out of 21 for biomarkers in general EVs versus 5 out of 16 in CNS‐originating EVs (Taha & Bogonwieski, 2023), indicating that the former approach has substantially less publication bias. Moreover, both methodologies suffered from substantially large heterogeneity, indicating that more rigor, standardization and independent validations across groups are needed.

**TABLE 5:** Comparison between bulk EVs versus CNS‐originating EVs for diagnosing Parkinson's disease (PD) from healthy controls using a bivariate and hierarchal summary receiver operating characteristics (HSROC) model. The sensitivity, specificity, pooled area under the curve (AUC) and partial AUC, focusing on a specific range of false positive rates (FPR), are obtained using the bivariate model. The diagnostic odds ratio (DOR) is obtained from the HSROC model. EV — Extracellular vesicles. CNS — Central nervous system. SE—Standard error.

EV source	Mean sensitivity (95% CI)	Mean specificity (95% CI)	Pooled AUC (partial AUC))	Mean DOR ± SE (95% CI)
**General EVs**	84.4% (77.7–90.7%)	79.1% (72.5–84.0%)	0.852 (0.672)	21.6 ±1.3 (12.0–38.9)
**CNS‐originating EVs**	72.5% (64.4–79.3%)	75.9% (69.2–81.4%)	0.800 (0.692)	8.3 ± 2.1 (5.0–13.8)

We also noted that only one study involving biomarkers in CNS‐originating EVs attempted to differentiate patients with PD versus HCs, while also employing general EVs for the ROC analysis (Yan et al., 2022). The rationale for the omission of such biomarkers in general EVs for diagnosis before transitioning to CNS‐originating EVs remains unclear. Notably, the isolation of CNS‐originating EVs is significantly more intricate, time‐consuming and labour‐intensive.

## 4 DISCUSSION

The absence of clear and exact biomarkers for the definitive antemortem diagnosis of parkinsonian disorders, including PD, MSA, DLB, PSP, and CBS frequently results in misdiagnoses, negatively affecting patients' access to suitable and prompt treatment (Baumann, 2012; Rizzo et al., 2016; Schrag et al., 2002). This issue is further compounded by the inability to predict prodromal disease conversion (e.g., RBD) to one of the three synucleinopathies; PD, MSA and/or DLB (Dauvilliers et al., 2018). This situation is unsettling for patients, who face uncertainty about their health and future as well as for physicians who aim to deliver the best care.

EVs are tiny vesicles believed to be released by all cells, serving as carriers of cell‐state‐specific messages to both neighbouring and distant cells. They have gained attraction as a popular source for biomarker discovery for parkinsonian disorders. While few meta‐analyses examined the utility of biomarker concentrations in EVs (Nila et al., 2022) and/or CNS‐originating EVs (Taha & Ati, 2023; Taha & Bogoniewski, 2023), no study to date has examined the diagnostic accuracy of biomarkers in general EVs isolated from bodily fluids for parkinsonian disorders.

The current meta‐analysis included 21 studies encompassing 1285 patients with PD, 24 with MSA, 105 with DLB, 99 with PSP, 101 with RBD, and 783 HCs. Because the number of studies was low (*n* ≤ 3) for differentiating parkinsonian disorders among each other or MSA, DLB, PSP, CBS, or RBD from HCs, we only conducted meta‐analyses for patients with PD versus HCs. We also noted that no study included a PAF cohort. This glaring omission in the literature signifies an urgent need for researchers to broaden their focus, encompassing not just patients with PD, but also other parkinsonian disorders. Addressing this disparity is vital for clinicians, as it could significantly enhance the accuracy and scope of differential diagnoses.

Both the bivariate and HSROC models revealed moderate diagnostic accuracy of this approach in distinguishing patients with PD from HCs (Figure 2f). However, substantial heterogeneity and variability across the studies, possibly due to differences in isolation and quantification methodologies, populations, and media for EV isolation, caution the interpretation of such results and emphasize the need for further validation. Additionally, publication bias (Figures 2a–2f) was substantial. The trim‐and‐fill method estimated that there are at least two studies with low or null diagnostic accuracy missing (Figure 3f), suggesting a potential overestimation of the diagnostic test's efficacy.

In the analysis comparing the diagnostic metrics by CSF, plasma, and serum for differentiating patients with PD from HCs, CSF‐EVs demonstrated better overall diagnostic accuracy (Figure 4a), likely due to CSF's direct connection with the brain. Plasma‐EVs analysis (Figure 4b) revealed the lowest performance, with possible publication bias, while serum‐EVs analysis (Figure 4c) showed moderate diagnostic accuracy without bias. Though the highest diagnostic accuracy was obtained for CSF in the meta‐analysis, the small number of studies using CSF (*n* = 4) makes drawing definitive conclusions difficult. Despite these findings, the significant heterogeneity observed across the models emphasizes the need for further studies. The selection among CSF, plasma, and serum for isolating EVs for biomarker discovery often depends on the goal, considering factors like CSF's connection with the brain and the minimally invasive nature of plasma and serum collection as well as the isolation and quantification methodologies available to the researchers.

The use of biomarkers in CNS‐originating EVs isolated from the blood in differentiating parkinsonian disorders from one another or from HCs has been popular (Dutta et al., 2023). Though isolating CNS‐originating EVs is a complicated and labour‐intensive process, the hope was that they would provide a more accurate reflection of the brain's biochemistry due to their ability to carry cell‐state‐specific messages across the blood–brain barrier to the peripheral circulation (Shi et al., 2014).

However, a comparative analysis found that general EVs, which are simpler to isolate, demonstrated higher diagnostic accuracy than CNS‐originating EVs (Table 5). Despite the advantage of general EVs, both methods were marked by substantial publication bias and large heterogeneity, but general EVs showed less bias. Specifically, the trim‐and‐fill method estimated 2 out of 21 versus 6 out of 15 missing studies with low or null diagnostic accuracy in general EVs versus CNS‐originating EVs. The poor diagnostic performance of CNS‐originating EVs could be due to some of the CNS antibodies, especially those targeting neuronal EV populations (e.g., L1CAM), cross‐reacting with the α‐synuclein antibody used for quantification (Norman et al., 2021). Few other explanations include the very small number of CNS‐originating EVs existing in the blood, the use of polymer‐based precipitation kits, believed to provide a “dirty” and heterogeneous EV yield (Brennan et al., 2020), in the majority of CNS‐originating EVs in comparison to general EVs, lack of CSF usage for CNS‐originating EVs isolation and the need for high expertise and precision in performing reproducible immunoprecipitation of CNS‐originating EVs.

It is also important to note that the majority of studies have not provided sufficient details about pharmacological treatments, including their type, dosage, and administration duration, which could influence the biomarkers measured in EVs. Also, if a patient passes away, studies should include any discrepancies between the antemortem and postmortem clinical diagnosis. Furthermore, there is a lack of data regarding participants' demographics and comorbid health conditions present, important factors that could influence the biomarker concentrations found in EVs. Lastly, researchers must not overlook the influence of preanalytical factors (Taha, 2023) and should document them thoroughly either in the methods or by utilizing the EV‐TRACK platform (Van Deun et al., 2017). These include the patient's fasting state before drawing blood, the specific time of day the blood is collected, how long the collection takes, the gauge of the needle used, the exact procedure and time taken to separate the blood components and the type of container or anticoagulant molecule used. Other critical details include how the sample is transported, whether the collection tube is kept upright or on its side, the method of centrifugation, the number of freeze/thaw cycles, the steps taken to deplete platelets, the storage conditions such as duration and temperature, treatments to remove the coagulation factors (e.g., thrombin), the method used for lysing the EVs and any processes used to freeze EVs or their lysates. All these elements must be rigorously recorded to ensure the integrity and reproducibility of the study. Because users may handle the samples used for EV isolation and subsequent biomarker quantification differently, researchers must indicate the number of persons involved in handling the samples as well as conduct subanalyses by the user. Finally, as opposed to singular studies, meta‐analyses like the one presented here often paint a markedly different picture, one that can challenge and even contradict the conclusions of individual studies. By integrating data across a spectrum of studies, the meta‐analysis can reveal underlying trends and discrepancies that single studies may not detect, sometimes casting doubt on upcoming diagnostic approaches. This comprehensive approach prompts a re‐evaluation of the evidence base and serves as a catalyst for more rigorous and standardized methodologies, ensuring that clinical practice is truly informed by the best available evidence, even when it overturns established beliefs.

## 5 CONCLUSION

While the diagnostic accuracy of biomarkers in general EVs holds promise and surpasses CNS‐originating EVs in distinguishing patients with PD from HCs, this approach remains far from feasible for practical translational use. Key points from our meta‐analysis include: (1) An emphasis on the urgent need to diversify research beyond just PD, addressing the notable gap in the literature by focusing on other parkinsonian disorders; (2) Moderate diagnostic accuracy of biomarkers within EVs in differentiating PD patients from HCs, yet substantial heterogeneity and potential overestimation due to publication bias; (3) CSF‐EVs showing superior diagnostic accuracy compared to plasma and serum, albeit based on a limited number of studies; and (4) The counterintuitive finding that general EVs, simpler to isolate, offer higher diagnostic accuracy than the labour‐intensive CNS‐originating EVs. However, it is essential to acknowledge that both methods demonstrate substantial bias and heterogeneity. The focus of research groups should shift towards harmonizing the field by striving for reliable and accurate results. This can be achieved through intensive independent validations, standardization of preanalytical factors, and methodologies. Collaboration, sharing best practices, and rigorous scientific investigation hold the potential to move this area of research from the realm of theory to practical clinical applications. Current efforts by the International Society for Extracellular Vesicles (ISEV) (Thery et al., 2018) and others (Gomes & Witwer, 2022; Van Deun et al., 2017) aim toward more rigorous reporting and standardization to enhance accuracy and reproducibility of research utilizing EVs. Lastly, authors are encouraged to report their detailed methodologies using the EV‐TRACK platform (Van Deun et al., 2017) to allow for better reproducibility and accuracy.

## CONFLICT OF INTEREST STATEMENT

The authors declare no conflict of interest.

## Supporting information

Supporting Information
