# Supplementary material for: Extracellular vesicles from bodily fluids for the accurate diagnosis of Parkinson's disease and related disorders: A systematic review and diagnostic meta‐analysis
Source: J Extracell Biol. 2023 Nov 13;2(11):e121. doi: 10.1002/jex2.121 (PMC11080888; doi:10.1002/jex2.121)
Supplement: Supplementary file 1 — Supporting Information [file JEX2-2-e121-s001.docx]

**Table S1.** Complete Search Strategy using PUBMED and EMBASE

| PUBMED | (((Parkinson's disease) AND (extracellular vesicle OR exosome)) AND (Diagnosis) |
| --- | --- |
| EMBASE | (parkinsons:ti,ab,kw AND disease:ti,ab,kw OR (multiple:ti,ab,kw AND system:ti,ab,kw AND atrophy:ti,ab,kw) OR (lewy:ti,ab,kw AND body:ti,ab,kw AND dementia:ti,ab,kw) OR (corticobasal:ti,ab,kw AND syndrome:ti,ab,kw) OR (progressive:ti,ab,kw AND supranuclear:ti,ab,kw AND palsy:ti,ab,kw)) AND (neuronal:ti,ab,kw AND extracellular:ti,ab,kw AND vesicles:ti,ab,kw OR evs:ti,ab,kw OR exosomes:ti,ab,kw OR (oligodendrocyte:ti,ab,kw AND extracellular:ti,ab,kw AND vesicles:ti,ab,kw) OR (oligodendrocyte:ti,ab,kw AND evs:ti,ab,kw) OR (astrocyte:ti,ab,kw AND extracellular:ti,ab,kw AND vesicles:ti,ab,kw) OR (astrocyte:ti,ab,kw AND evs:ti,ab,kw) OR (microglia:ti,ab,kw AND extracellular:ti,ab,kw AND vesicles:ti,ab,kw) OR (microglia:ti,ab,kw AND evs:ti,ab,kw)) AND (cns:ti,ab,kw OR brain:ti,ab,kw OR (central:ti,ab,kw AND nervous:ti,ab,kw AND system:ti,ab,kw) OR ('cns originating':ti,ab,kw AND evs:ti,ab,kw) OR ('cns derived':ti,ab,kw AND evs:ti,ab,kw)) |

**Table S2.** Rubric for QUADAS-2.

| DOMAIN | DOMAIN 1  Patient selection | DOMAIN 2  Index test(s) | DOMAIN 3  Reference standard | DOMAIN 4  Flow and timing |
| --- | --- | --- | --- | --- |
| Description | Describe methods of patient selection.  Describe included patients (prior testing, presentation, intended use of index test and setting). | Describe the index test and how it was conducted and interpreted. | Describe the reference standard and how it was conducted and interpreted. | Describe any patients who did not receive the index test(s) and/or reference standard or who were excluded from the 2x2 table (refer to flow diagram).  Describe the time interval and any interventions between index test(s) and reference standard. |
| Signaling questions (*yes/no/unclear*) | Was a case-control design avoided?  Was a consecutive or random sample of patients enrolled?  Did the study avoid inappropriate exclusions? | Were the index test results interpreted without knowledge of the results of the reference standard?  If a threshold was used, was it pre-specified? | Is the reference standard likely to correctly classify the target condition?  Were the reference standard results interpreted without knowledge of the results of the index test? | Was there an appropriate interval between index test(s) and reference standard?  Did all patients receive a reference standard?  Did patients receive the same reference standard?  Were all patients included in the analysis? |
| Overall judgement | Included studies only were considered eligible if they measured any extracellular vesicle (EV) related biomarker isolated from the bodily fluids of patients with at least Parkinson’s disease (**low risk of bias**) and one other parkinsonian disorder or healthy controls (HCs)  Unclear risk of bias was considered in the absence of information on consecutive patient enrollment.  High risk of bias was based on the absence of information on consecutive patient enrollment and any unexplained or suspected exclusions. | Even though knowledge of the diagnosis may affect the interpretation of the diagnostic test results, measuring α-syn in nEVs or oEVs is an objective method that should not be influenced by the diagnosis.  This is considered a low risk of bias, even if blinding was not used. | To diagnose PD, the standard used was the United Kingdom Parkinson's Disease Society Brain Bank or the MDS clinical diagnostic criteria were used. To diagnose MSA, the second consensus statement on the diagnosis of multiple system atrophy was used To diagnose DLB, the fourth consensus report of the DLB consortium was used. To diagnose PSP, the NINDS-SPSP International workshop or the movement disorder society criteria were used To diagnose CBS, the criteria for the diagnosis of corticobasal degeneration were used. The clinical diagnoses were established before the index test (low risk of bias).  If the diagnosis was based on symptoms/signs without consultation of the diagnostic criteria listed above, the study was rated as “unclear risk of bias”. | All patients were classified according to the appropriate diagnostic criteria (see Domain 3).  Low risk of bias was considered if all the questions were answered “yes”.  Unclear risk of bias was considered if they did not cover the time interval between clinical diagnosis and index test.  High risk of bias was considered if the study excluded any of the participants from the analysis. |
| Concerns regarding applicability  (*High/low/unclear*) | Are there concerns that the included patients do not match the review question? | Are there concerns that the index test, its conduct, or interpretation differs from the review question? | Are there concerns that the target condition as defined by the reference standard does not match the review question? | Could the patient flow have introduced bias? |
| Applicability:  Overall judgement | As reported in the inclusion and eligibility criteria, the clinical diagnoses for the diseases were based on established diagnostic criteria (see Domain 3). Therefore, all studies were rated as “low concern/high applicability” | If the protein concentrations were determined using a standard calibration curve, the risk of bias was considered low. If this information was not provided, the risk of bias was deemed unclear.  The usage of in-house developed tests was considered “high concern/low applicability.”  If RNA concentrations were determined using a highly specific technique such as qPCR, the risk of bias was deemed low.  If this information provided, it was deemed unclear. If the study used any other method, it was deemed “high concern/low applicability.” | Because all the studies used internationally recognized criteria for their assessments, the risk of bias was considered low. | NA |

**Table S3.** Risk of Bias assessment according to the QUADAS-2 per study included in the meta-analyses. L (GREEN) = low risk of bias. H (RED) = high risk of bias. U (ORANGE) = unclear risk of bias.

|  | Bias | | | | Applicability concern | | | | |  |
| --- | --- | --- | --- | --- | --- | --- | --- | --- | --- | --- |
|  | Patient selection | Index text | Reference standard | | | Flow and timing | Patient selection | Index text | Reference standard | |
| **Studies** | | | | | | | | | | |
| Gui et al. 2015 | U | L | | L | | L | L | L | L | |
| Stuendl et al. 2016 | U | L | | L | | L | L | L | L | |
| Dos Santos et al. 2018 | U | L | | L | | L | L | L | L | |
| Hong et al. 2021 | U | L | | L | | L | L | L | L | |
| Tong et al. 2022 | U | L | | L | | L | L | L | L | |
| Vacchi et al. 2020 | U | L | | L | | L | L | L | L | |
| Chung et al. 2021a | U | L | | L | | L | L | L | L | |
| Chung et al, 2021b | U | L | | L | | L | L | L | L | |
| Shim et al. 2021 | U | L | | L | | L | L | L | L | |
| Zheng et al. 2021 | U | L | | L | | L | L | H | L | |
| Stuendl et al. 2021 | U | L | | L | | L | L | L | L | |
| Lucien et al. 2022 | U | L | | L | | L | L | H | L | |
| Xie et al. 2022 | U | L | | L | | L | L | L | L | |
| Yan et al. 2022 | U | L | | L | | L | L | H | L | |
| Cao et al. 2017 | U | L | | L | | L | L | L | L | |
| Barbagallo et al. 2019 | U | L | | L | | L | L | L | L | |
| Manna et al. 2021 | U | L | | L | | L | L | L | L | |
| Wang et al. 2019 | U | L | | L | | L | L | H | L | |
| Hadisurya et al. 2023 | U | L | | L | | L | L | H | L | |
| Cao et al. 2019 | U | L | | L | | L | L | L | L | |
| Cao et al. 2020 | U | L | | L | | L | L | L | L | |

**FIGURE S1.** Diagnostic accuracy of biomarkers in cerebrospinal fluid (CSF) extracellular vesicles (EVs) for the differential diagnosis of patients with Parkinson’s disease (PD) from healthy controls (HCs). **(A-E)** Univariate Forest plots for sensitivity, specificity, diagnostic odds ratio (DOR), positive (posLR) and negative (negLR) likelihood ratios, respectively.
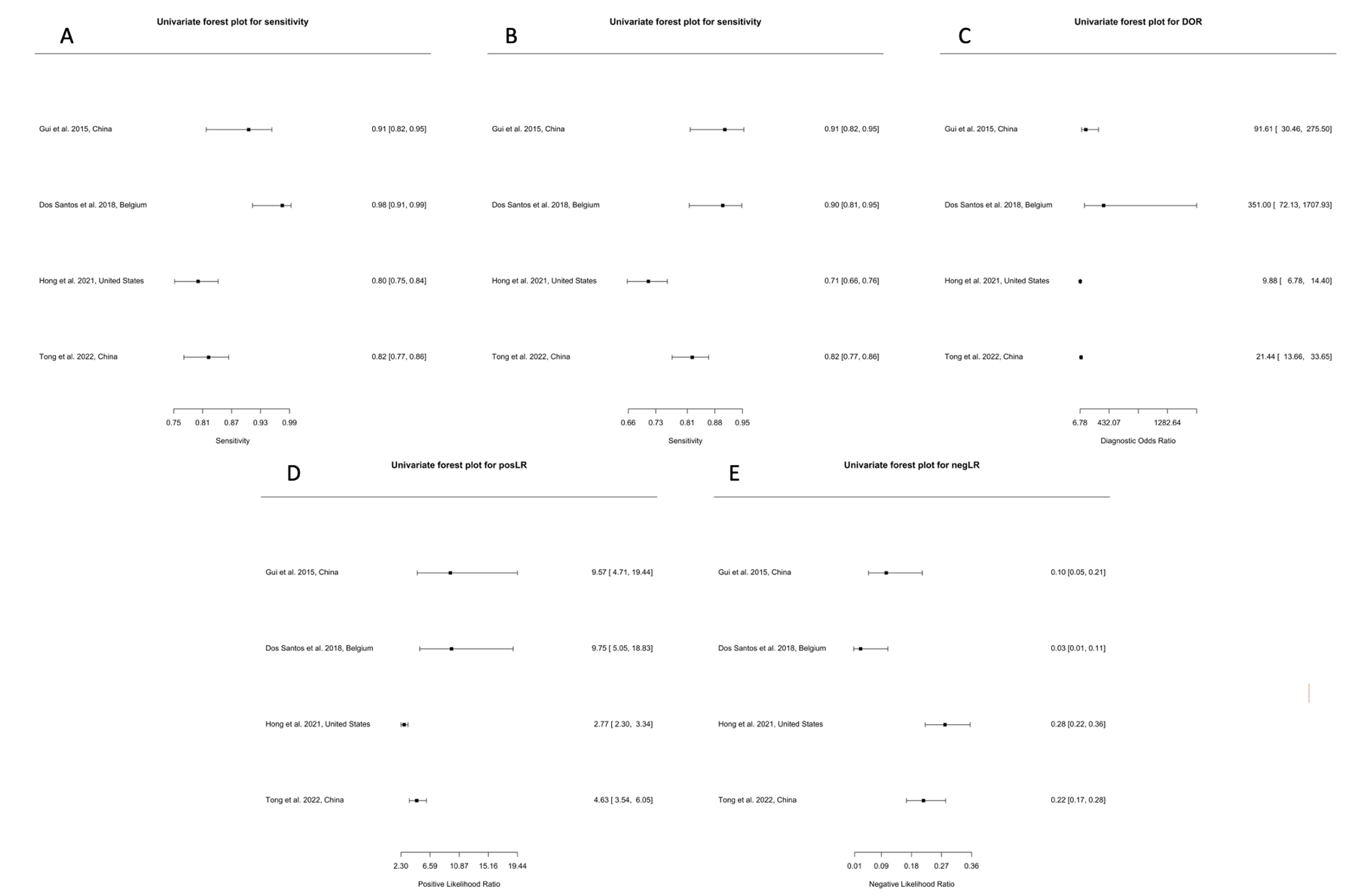


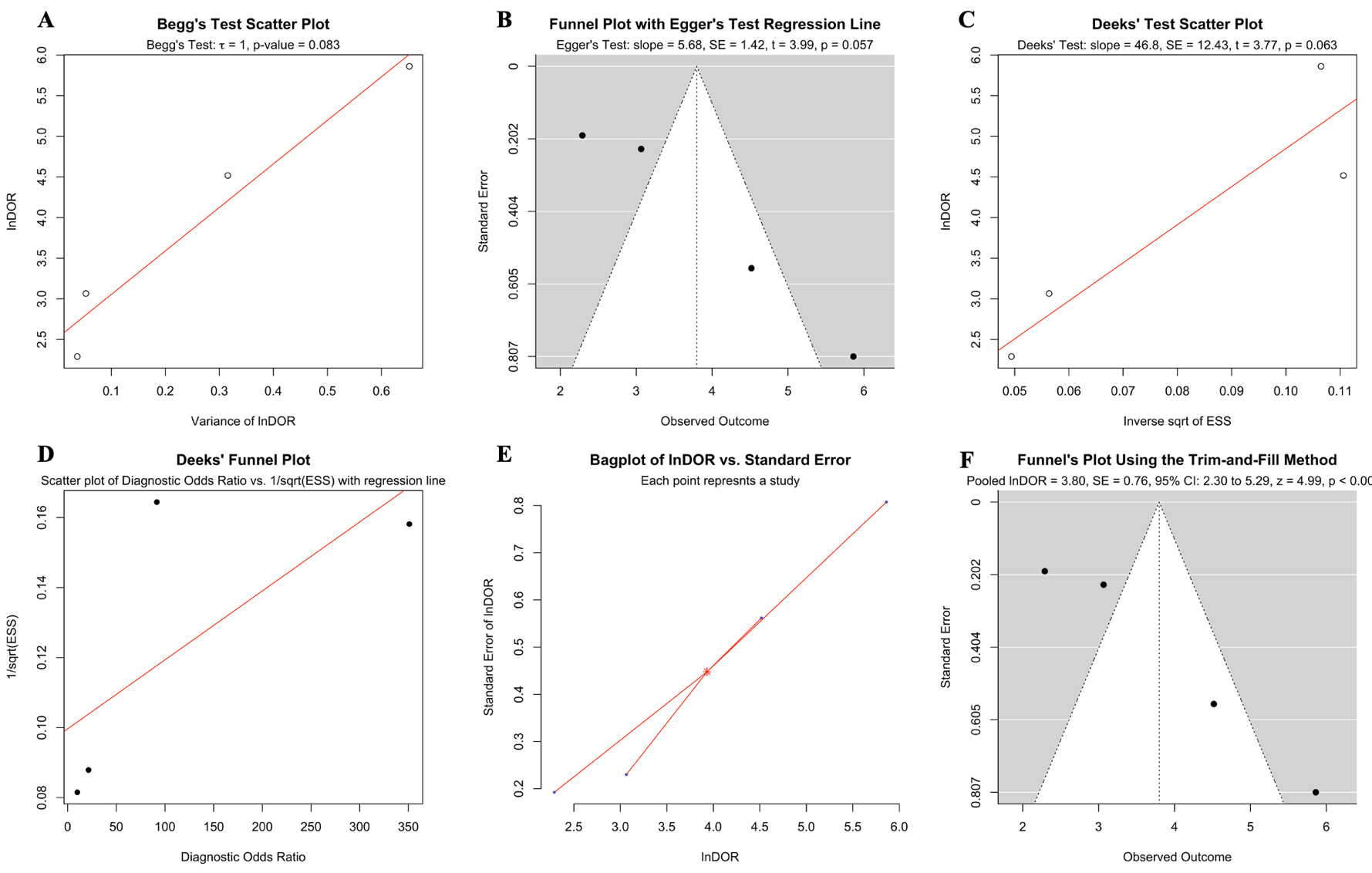


**FIGURE S2.** Publication bias assessed using **(A)** Begg’s correlation, **(B)** Egger’s regression, **(C)** Deek’s regression, **(D)** Deek’s funnel plot, **(E)** A bagplot and **(F)** Funnel plot after application of the trim-and-fill method for biomarkers in cerebrospinal fluid (CSF) extracellular vesicles (EVs) for the differential diagnosis of patients with Parkinson’s disease from healthy controls (HCs). Collectively, they suggested no presence of publication bias.


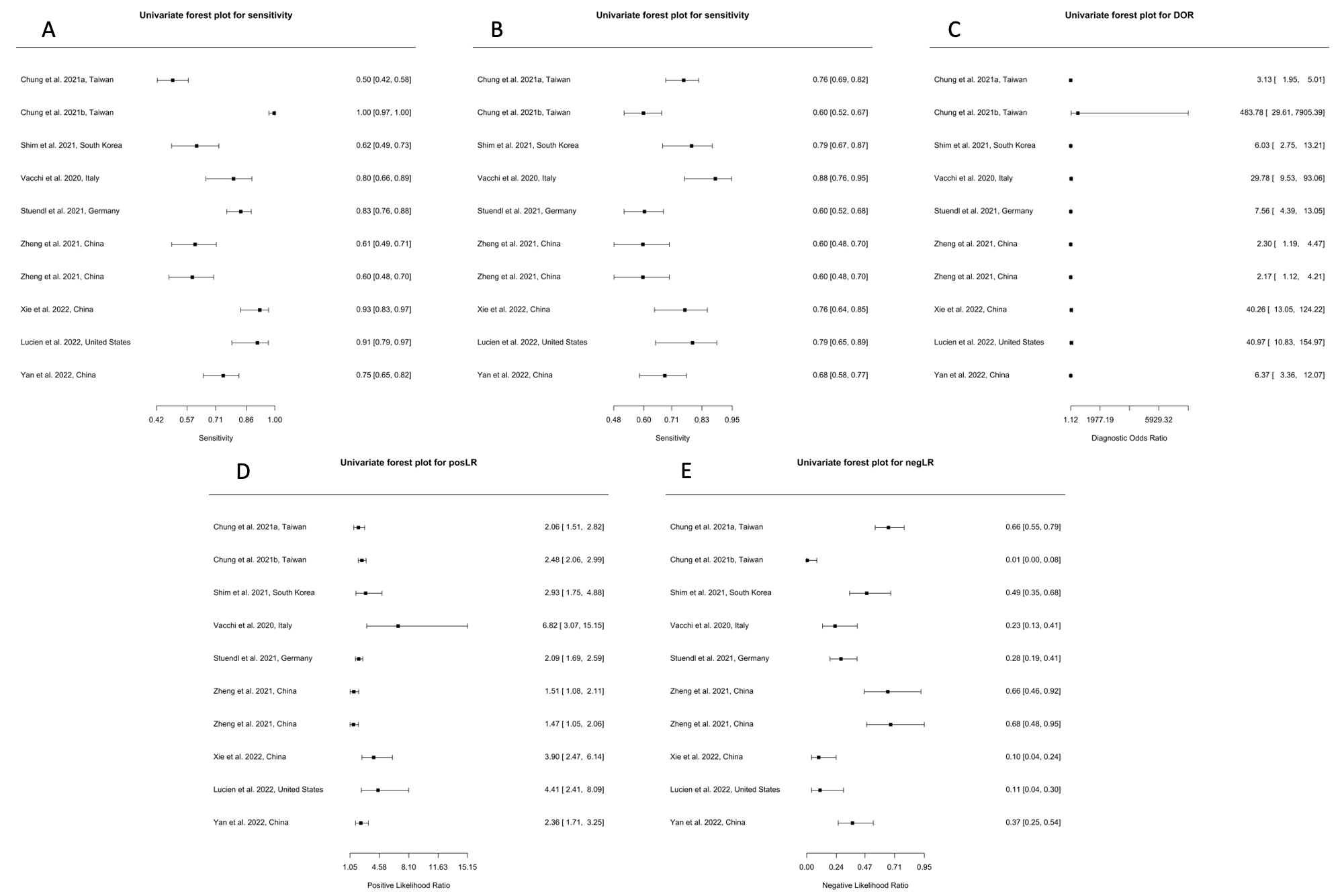


**FIGURE S3.** Diagnostic accuracy of biomarkers in plasma extracellular vesicles (EVs) for the differential diagnosis of patients with Parkinson’s disease (PD) from healthy controls (HCs). **(A-E)** Univariate Forest plots for sensitivity, specificity, diagnostic odds ratio (DOR), positive (posLR) and negative (negLR) likelihood ratios, respectively.


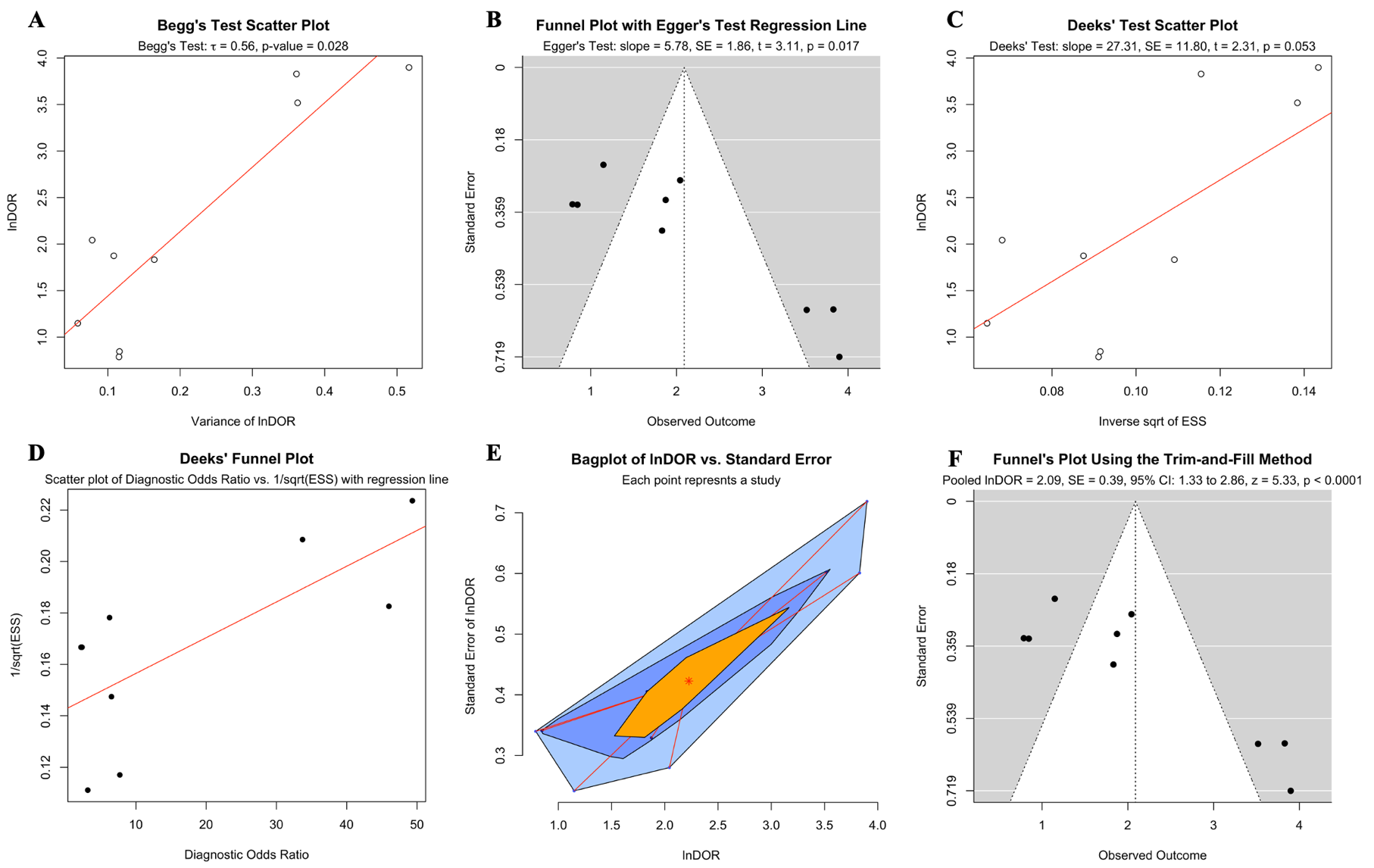


**FIGURE S4.** Publication bias assessed using **(A)** Begg’s correlation, **(B)** Egger’s regression, **(C)** Deek’s regression, **(D)** Deek’s funnel plot, **(E)** A bagplot and **(F)** Funnel plot after application of the trim-and-fill method for biomarkers in plasma extracellular vesicles (EVs) for the differential diagnosis of patients with Parkinson’s disease from healthy controls (HCs). Collectively, they suggested the possible presence of publication bias.


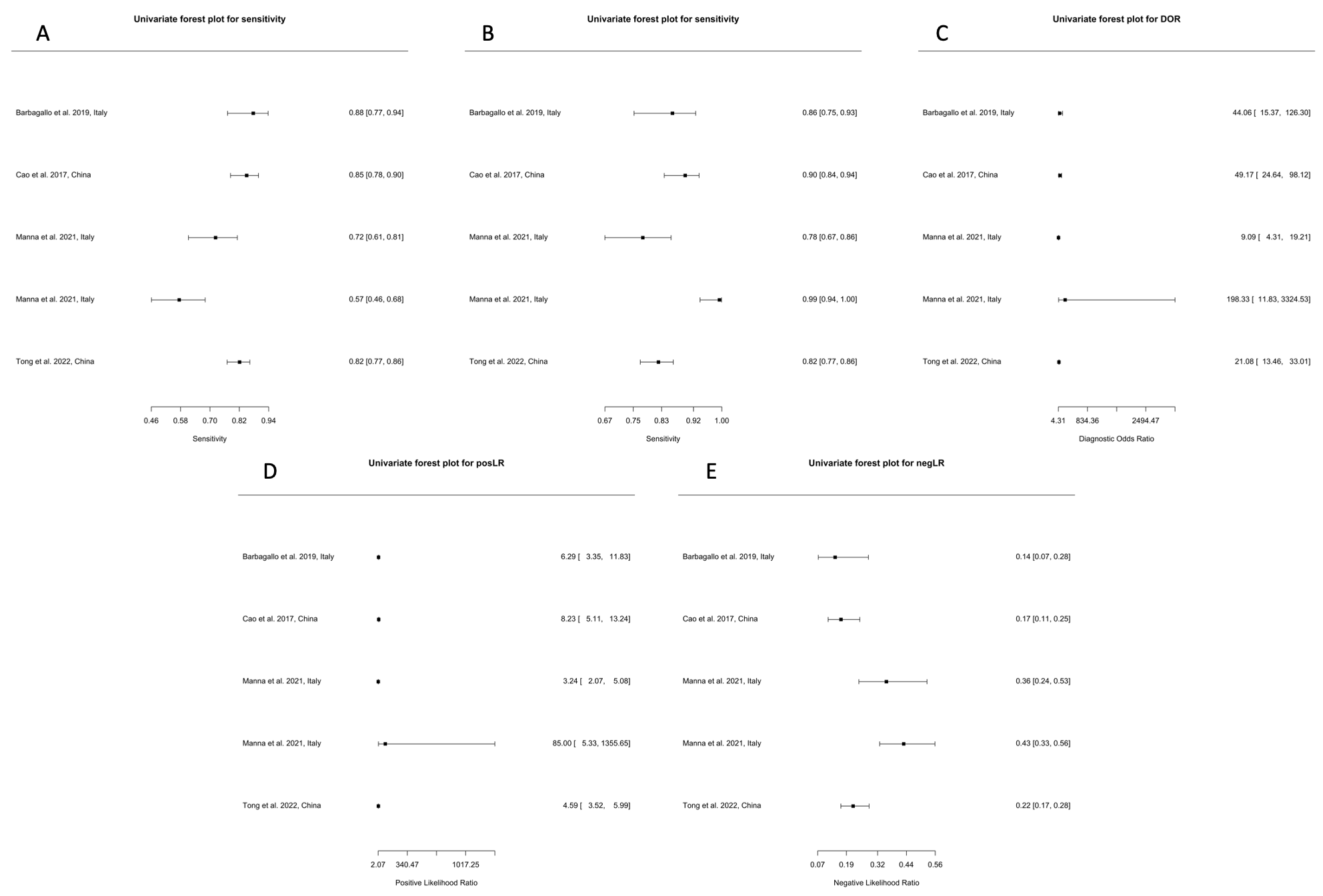


**FIGURE S5.** Diagnostic accuracy of biomarkers in serum extracellular vesicles (EVs) for the differential diagnosis of patients with Parkinson’s disease (PD) from healthy controls (HCs). **(A-E)** Univariate Forest plots for sensitivity, specificity, diagnostic odds ratio (DOR), positive (posLR) and negative (negLR) likelihood ratios, respectively.


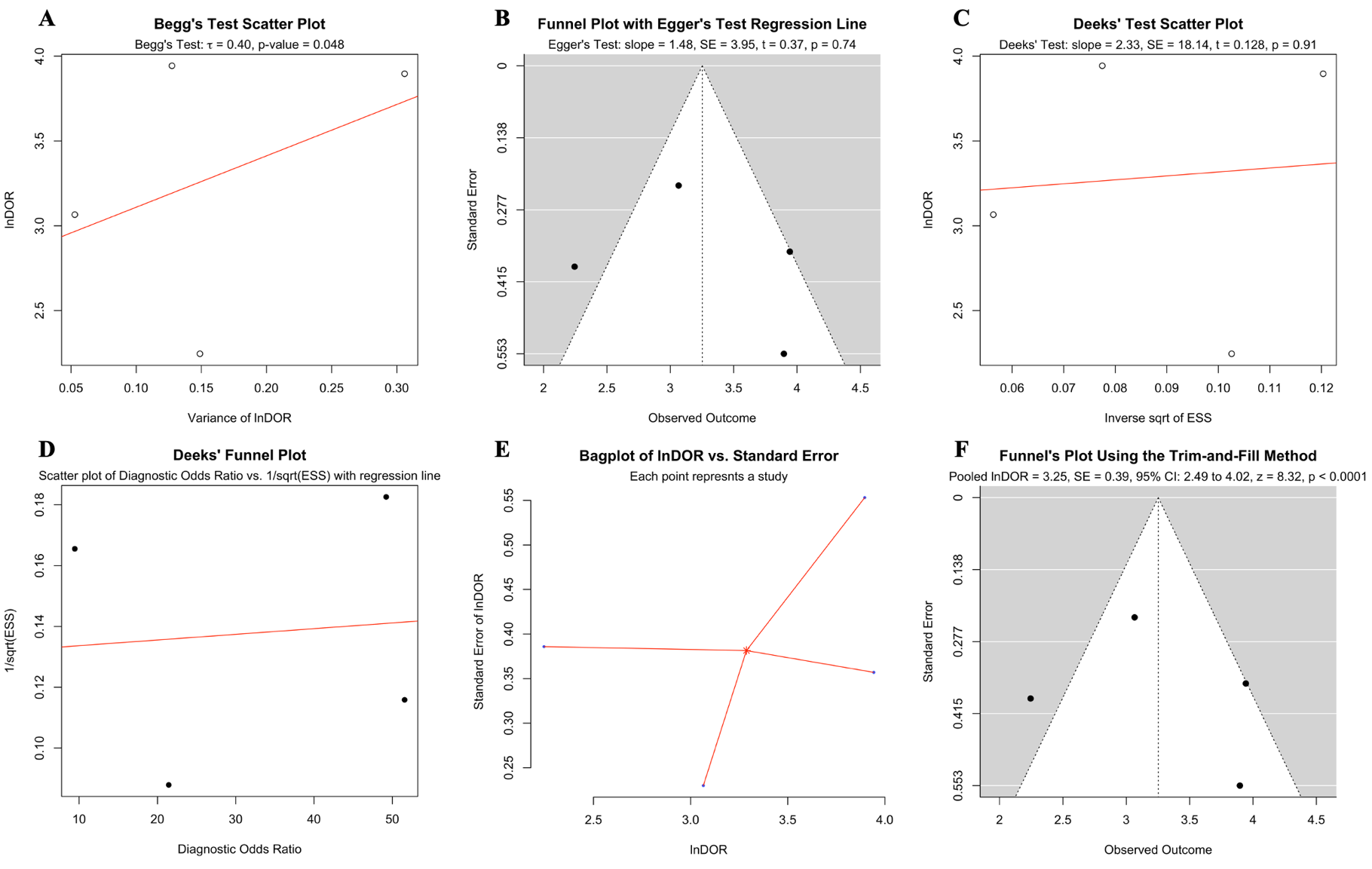


**FIGURE S6.** Publication bias assessed using **(A)** Begg’s correlation, **(B)** Egger’s regression, **(C)** Deek’s regression, **(D)** Deek’s funnel plot, **(E)** A bagplot and **(F)** Funnel plot after application of the trim-and-fill method for biomarkers in serum extracellular vesicles (EVs) for the differential diagnosis of patients with Parkinson’s disease from healthy controls (HCs). Collectively, they suggested no presence of publication bias.
